# Live-Cell Imaging of Telocyte-like Cells in Primary Liver Cell Cultures

**DOI:** 10.3390/cells15161476

**Published:** 2026-08-18

**Authors:** Nikolaus Bresgen, Hubert H. Kerschbaum

**Affiliations:** Department of Biosciences and Medical Biology, University of Salzburg, A-5020 Salzburg, Austria; hubert.kerschbaum@plus.ac.at

**Keywords:** telocytes, primary hepatocytes, exploratory behavior, macrophages

## Abstract

Telocytes are small mesenchymal cells with unknown hepatic functions. In primary cultures of rat hepatocytes and non-parenchymal liver cells (NPLC), we observed, at a very low incidence, small-sized, highly motile cells. Because these cells fulfill ultrastructural/morphological ‘telocytes hallmarks’, foremost their minuteness and the presence of very thin telopodia, we refer to them as hepatic telocyte-like cells (hTCLs). Unique among hepatic cells and beyond these telocyte characteristics is their high motility and pronounced cell plasticity. Surprisingly, hTCLs display an exceptional exploratory behavior by moving along/between cell borders as well as beneath cells. Their fast motility apart, hTCLs share structural and behavioral similarities with Kupffer cells/macrophages in NPLC cultures, suggesting a relationship between both cell types that needs to be addressed by further investigation. In EGF/insulin-treated hepatocyte cultures, hTCLs’ motility declines, and the cells adopt an extremely small cell body with very long processes. In cultured hepatocyte monolayers, hTCLs associate with dividing hepatocytes, especially during advanced mitosis (cytokinesis). In conclusion, our observations demonstrate the presence of a small population of highly mobile, telocyte-like cells in liver-derived cell cultures, and a correlative association between hTCLs and hepatocytes, hypothetically accounting for an integrative function of this cell type.

## 1. Introduction

As defined by Popescu and Faussone-Pellegrini in 2010, telocytes represent interstitial Cajal-like cells (ICLC) of mesenchymal origin that can be found in several organs including the gut, liver, pancreas, blood vessels, heart, mammary gland, and placenta, as well as the male and female reproductive tract [1,2].

Telocytes are marked by a small, piriform- to spindle-shaped cell body (6 to 16 µm in length); a high nucleus-to-cell/cytoplasm ratio (about 25% of the telocyte is covered by the nucleus); and, most specifically, the presence of extremely long (up to several hundred µm), delicate telopodia of less than 0.2 µm in diameter, which display characteristic dilations, termed podoms [1,3,4]. Due to their size, telopodia fall below the resolution limits of light microcopy and may not be seen upon inspection via brightfield or phase contrast. This renders telocytes different from other process-forming cells such as macrophages, which display markedly thicker uropodia that are readily visible by light microscopy even at lower magnification. Moreover, processes like macrophage uropodia, as well as others formed in cells such as fibroblasts and myofibroblasts, emerge from the cell body as a thickened structure that gradually tapers into a thin cell process. This does not apply to telocytes, where the telopodium emerges from the cell soma in a rather sharply demarcated fashion [1,5].

Under functional aspects, telocytes have been shown to be able to form distinct contacts with neighboring cells, also termed stromal “synapses” [6]. Together with other telocyte properties, the presence of stromal synapses allows the formation of networks between telocytes and neighboring cells, which may play a role in several physiologic contexts including organization of the extracellular matrix (ECM), regulation of cell proliferation and differentiation, and mediation of neurotransmitter-associated activities such as those discussed for the function of telocytes in gut motility regulation [4]. Telocytes may also establish distinct cell–cell contacts with resident macrophages of the gut mucosa, which serves nutritional contexts [7]. Moreover, telocytes are considered as important players in developmental contexts, wound healing, tissue repair, and regeneration [8,9,10,11,12,13,14]. Finally, recent findings suggest that telocytes also participate in the regulation of stem cell activity and texturing of stem cell niche microenvironments [15].

In the liver, electron microscopy has revealed that telocytes locate exclusively in the Space of Disse and in contrast to Kupffer cells are absent from liver sinusoids [2]. The same study has also shown that telocytes, by the presence of telopodia, differ from hepatic stellate cells (HSCs), which also reside in the Space of Disse. Xiao and co-workers have further demonstrated the immunoreactivity of hepatic telocytes for vimentin, CD34, c-kit/CD117, and PDGFRα (platelet-derived growth factor receptor α). Among these antigens, CD34 and PDGFRα are considered the most reliable telocyte markers, although their expression may depend on tissue- and organ-specific contexts [4]. This is of relevance, as hepatic telocytes may participate in wound healing and liver regeneration [1,9,16]. It is sound to consider that hepatic telocytes participate in early/immediate phases of wound healing and may also be involved in tissue reorganization during the compensatory growth of hepatocytes. Indeed, Wang and co-workers have shown that after partial hepatectomy, telocyte numbers are shifted concomitantly to hepatocyte proliferation [16]. Outside the liver, telocytes have been shown to express EGF and TGFß1 [11,17,18,19], two essential growth factors that are critical to the compensatory growth of hepatocytes [20,21]. Hence, potential telocyte–hepatocyte interactions governing liver parenchyma replenishment upon liver injury are of considerable interest in elucidating the physiological role of hepatic telocytes in liver regeneration. Irrespective of this evidence, we are just beginning to understand the implications of telocytes in liver physiology. An actual (16 July 2026) search in PubMed using the term “telocytes liver” returned 42 matches (for comparison: the search for “telocytes” returned 763 matches). Excluding reviews, book chapters, and matches related to other tissues, 11 articles provide original experimental data on liver telocytes. Almost all of these articles have investigated the presence of telocytes in liver tissue sections, and only one report has recently become available on primary telocyte cultures established from newborn mice [22].

We identified very small, unusually fast-moving cells in pure primary rat hepatocyte cultures established from rats by the two-step in situ perfusion technique [23]. By ultrastructural and morphological criteria, these cells fulfill the criteria defined by Popescu and co-workers as ‘telocyte hallmarks’ [1,3]. Accordingly, we termed these cells hepatic telocyte-like cells (hTCLs). We used live-cell imaging (LCI) of primary cultures of parenchymal rat hepatocytes and mixed cultures of non-parenchymal liver cells (NPLC) to examine interactions between hTCLs, hepatocytes, and NPLCs. Here, we will report that hTCLs are very rare in both cultures but nevertheless represent a unique telocyte-like cell type, which displays an exceptional migratory behavior that has not yet been described for primary liver cell cultures.

## 2. Materials and Methods

**Animals.** Experiments were conducted in compliance with ethical regulations as defined by national (Austrian national animal experimentation law 2012) and European guidelines (Directive 2010/63/EU of the European Parliament on the protection of animals used for scientific purposes). According to Directive 2010/63/EU “*killing of animals solely for the use of their organs and tissues*” is not considered as a ‘procedure’ or ‘animal experiment’. As in our study, animals were killed solely for the purpose of organ use/cell isolation (i.e., for establishing primary cell cultures), so no permissions were required. Adult 8–12 weeks old female Fischer 344 rats (obtained from Charles River, Germany) were kept in the certified central husbandry of the University of Salzburg in a temperature and humidity-controlled room with a 12 h light–dark cycle. Food and water were provided ad libitum. Animals were allowed to acclimate for at least two weeks prior to hepatocyte isolation.

**Materials.** MEM with Earle’s salts and nonessential amino acids was obtained from Invitrogen, Vienna, Austria. Antibiotics were obtained from Lonza through Szabo-Scandic, Vienna, Austria. Collagenase and other cell culture chemicals—unless otherwise specified—were obtained from Sigma-Aldrich, Vienna, Austria. Recombinant human EGF was obtained from Sanova, Vienna, Austria. Percoll^®^ was obtained from GE Healthcare, Vienna, Austria. Plasticware was obtained from Sarstedt and Falcon/Beckton Dickinson, Austria. Petri dishes for live-cell imaging were obtained from IBIDI (Munich, Germany).

**Culture medium (MEM) used for culturing primary parenchymal rat hepatocytes.** Serum-free minimum essential medium (Invitrogen # 41500-018), supplemented with aspartate (0.2 mM), serine (0.2 mM), pyruvate (10 mM), penicillin (100 U), and streptomycin (100 µg/mL) was used, indicated as MEM in the text. In several experiments, MEM supplemented with EGF (40 ng/mL) and insulin (0.9 µg/mL) was used for stimulating hepatocyte proliferation.

**Isolation of primary parenchymal hepatocytes.** Parenchymal rat hepatocytes were isolated by the two-step in situ collagenase liver perfusion technique described by Michalopoulos et al. [23]. After washing the freshly isolated cells twice in calcium-free buffer (142 mM NaCl, 6.7 KCl, 10 mM Hepes, pH 7.4), low-speed centrifugation was employed (44× *g*, 10 min), which yields a sedimented fraction of parenchymal hepatocytes with high purity (>98%), and the cell numbers and viability (Trypan Blue exclusion assay) of the cell preparations were determined. Only hepatocyte preparations with a viability ≥ 90% were used for experimental work. After cell isolation, dead and remaining non-parenchymal cells were removed from the initial cell preparation by employing a self-generating Percoll^®^ density gradient, yielding high purity as described previously [24].

**Establishing mixed cultures of non-parenchymal rat hepatocytes (NPLC).** As outlined above, parenchymal hepatocytes are quantitatively separated from the collected whole liver cell mass upon low-speed centrifugation (44× *g*, 10 min). For collecting the NPLC fraction, the supernatant obtained after the first centrifugation containing NPLCs, a small number of parenchymal hepatocytes and some adventitious blood cells are centrifuged at 240× *g*/10 min, and the sedimented cells are resuspended in 5 mL DMEM/10% FCS. Then, primary cultures are established by inoculating the cells at a dilution of 1:5 (in DMEM/10% FCS) in T25 tissue culture plastic flasks. After initial incubation for 2 h, the cultures were washed 2–3 times to remove blood cells and other non-adherent cells. Thereafter, the NPLC cultures were incubated for 1–2 weeks until the cell monolayer reached confluence. The culture medium (DMED/10%FCS) was changed every 3–4 days. During these medium changes, remaining blood cells were removed. Primary parenchymal hepatocytes were sporadically seen at early time-points of primary NPLC cultures but disappeared at early culture and were not detectable in confluent NPLC cultures. NPLC cultures were propagated for several weeks by splitting 1:5 upon reaching confluence.

**Live-cell imaging (LCI).** LCI experiments were performed using a NIKON Biostation IQ (Nikon Corporation, Tokyo, Japan) under standard culture conditions (36 °C, 5% CO_2_, 95% relative humidity). Parenchymal hepatocytes: Viable hepatocytes (1 × 10^5^) were plated in 3 mL of serum-free MEM on collagen-coated 35 mm IBIDI LCI-suitable Petri dishes. One hour after plating, the medium was exchanged for either fresh MEM or MEM supplemented with EGF (40 ng/mL) and insulin (0.9 µg/mL). Primary NPLC cultures: Confluent NPLC cultures were washed twice, detached using trypsin/EDTA (applied for 5–10 min), and the cells plated at a dilution of 1:5 in IBIDI LCI-suitable Petri dishes without a further medium change.

**LCI monitoring and video editing.** LCI was performed over a total time interval of 72 h (parenchymal hepatocytes) or 24 h (NPLCs) at a time resolution of one frame per min (except in one video with a resolution of one frame/2 min). Appropriate video sequences were selected and extracted using the NIKON AR imaging software (Version 4.40.00). Selected individual video frames were extracted as high-resolution TIFF files for preparing the figures. For video presentation (Videos S1–S17), annotations were applied to the videos using Adobe Premiere Pro 2026. The high-resolution LCI videos were converted into H.264/mp4 compressed media format using the Adobe Media Encoder 2026. No image processing other than applying the *slightly sharpen algorithm* (NIKON AR imaging software) to compensate for compression-based loss of sharpness (“blur”) was applied to the LCI videos.

**LCI-based morphometric analysis**. Cell tracking was performed in primary cultures of parenchymal hepatocytes and NPLCs by measuring the movement distances of individual hTCLs between each two consecutive frames (i.e., within one min). Morphometric measurements of cell and nucleus size were also performed on selected frames. Morphometric measurements were performed using the NIKON AR imaging software. The results of the morphometric analysis are given in Table 1 and Tables S1 and S2. hTCL trajectories were annotated in the LCI videos by color-coded lines. For determining cell and nuclear size (Figure S2), nucleus areas were defined by applying morphometric tools. Selected areas were measured in µm^2^, and diameters were calculated from these areas as equivalent diameter (see Table S1).

**Scanning electron microscopy (SEM).** For SEM, 1 × 10^5^ viable cells were plated on 13 mm diameter collagen-coated plastic coverslips in 24-well-plates containing 1 mL serum-free MEM per well. After incubation for 1 h under standard culture conditions to allow cell attachment, the medium was changed for MEM supplemented with EGF (40 ng/mL) and insulin (0.9 µg/mL) and the cells were returned to the incubator. After 24 h, cells were fixed with 2.5% (*v*/*v*) glutaraldehyde in phosphate-buffered saline (PBS, pH 7.2–7.4) for 1 h, rinsed with PBS four times within 1 h, followed by dehydration in a graded series of ethanol (50–100% *v*/*v*). Subsequently, cells were critical-point dried (Baltec Critical Point Dryer LPD030, BAL-TEC AG, Balzers, Liechtenstein). Finally, the coverslips were glued onto pins, sputter-coated with gold using an Agar Sputter Coater (Agar Scientific Ltd., Stansted, UK). The cells were visualized using a CAMBRIDGE Stereoscan 250 scanning electron microscope (Cambridge Scientific Instruments Limited, Cambridge, UK).

**Statistics.** With respect to the very low numbers of hTCLs that were accessible to morphometric analysis within the captured video frames, data were analyzed by descriptive statistics, omitting any inferential statistics. IBM SPSS Statistics version 31.0.0.0 was used.

## 3. Results

### 3.1. hTCL Are Present in Primary Liver Cell Cultures

Isolation of parenchymal hepatocytes, subsequently referred to as hepatocytes, using the two-step in situ collagenase liver perfusion technique combined with Percoll enrichment yields highly pure hepatocyte cell suspensions. Notably, hepatocytes prepared by the two-step in situ collagenase perfusion method contain <1–2% non-parenchymal liver cells (NPLC) already after washing the initial cell pellet twice in buffer, and about 0.1% NPLC are found as contaminating cells in primary hepatocyte cultures after 48 h of serum-free cultivation [25,26]. The purity is substantially shifted further by adding the Percoll enrichment step. According to our experience, serum-free primary cultures established from such pure hepatocyte preparations are devoid of contaminating NPLCs.

Nevertheless, occasionally very small, fast-moving cells (Video S1), termed hTCLs (hepatic telocyte-like cells), can be observed in pure hepatocyte cultures as well as mixed cultures of NPLC by live cell imaging (LCI). Referring to cell size (10.9 ± 0.4 µm) and the nucleus-to-cell ratio (0.31 ± 0.03) (Table S2), hTCLs share high similarity with telocytes [1]. Of special relevance, like telocytes, hTCLs also contain telopodia, which are barely detectable in LCI due to the resolution limits of light microscopy (outlined in Section 3.5 and Section 3.6). Moreover, hTCL may appear piriform- to spindle-shaped (outlined in Section 3.5 and Section 3.8), as described for telocytes by Popescu and Faussone-Pellegrini. Finally, like telocytes, hTCLs are scarcely seen in liver-derived cell cultures. With an incidence of 1:124 (hepatocytes) and 1:29 (NPLC) (see Table S1), there is a high chance that hTCLs escape detection by routine microscopical inspection of living as well as fixed cell cultures.

**Figure 1:**
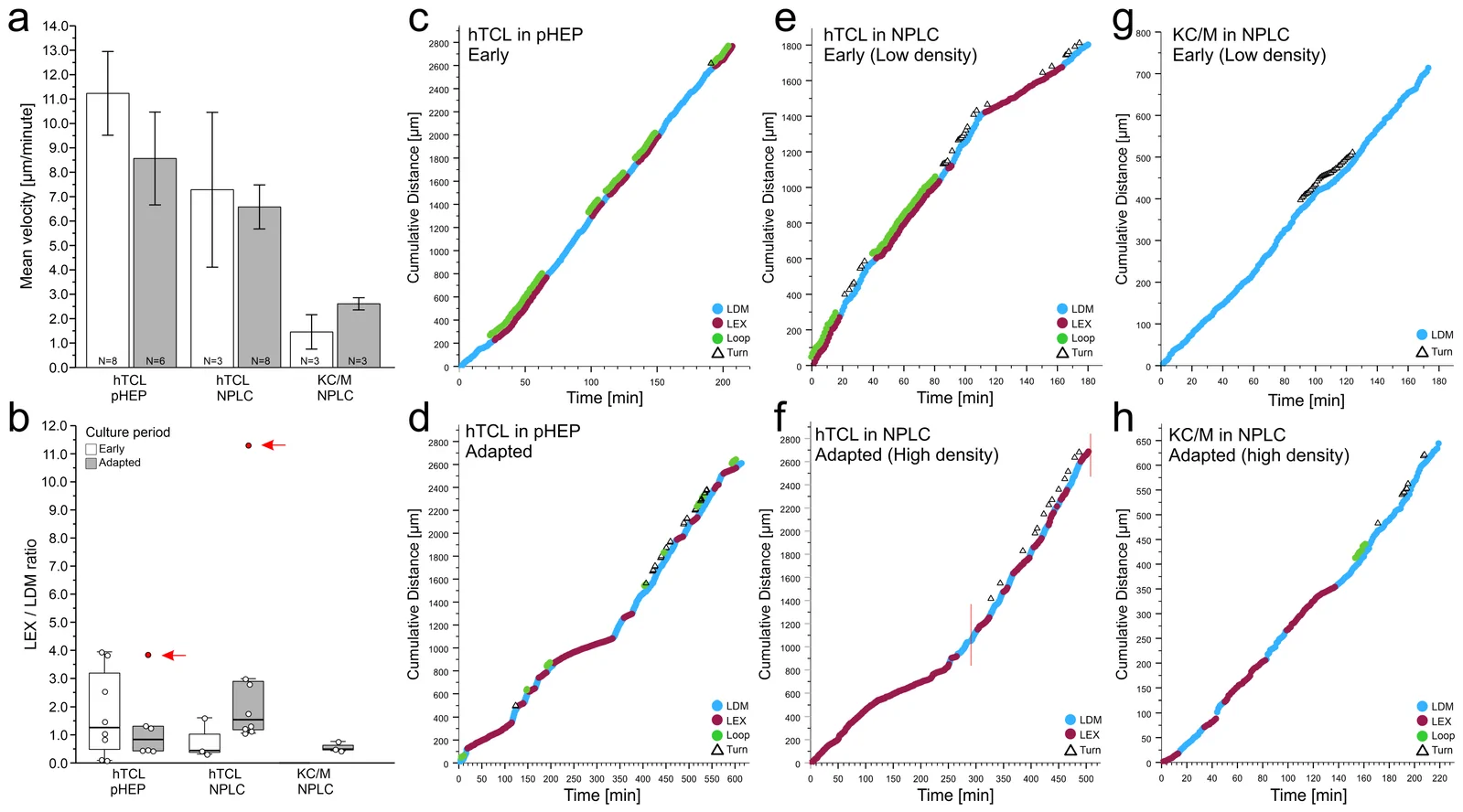
Analysis of hTCL and KC/macrophage trajectories monitored by LCI in primary cultures of rat hepatocytes (pHEP) and non-parenchymal rat liver cells (NPLC) during early and adapted culture stages. (**a**) Velocity (µm/min) of hTCL and KC movement. Bars represent the mean ± 2SEM of the movement velocities (open bars, early culture; gray bars, adapted cultures). The number of independent trajectories monitored by LCI is given in parentheses; corresponding data are given in Table 1 and Table S3. (**b**) LEX:LDM ratio for the hTCL and KC/macrophage trajectories in pHEP and NPCL cultures. The LEX:LDM ratio is calculated as the ratio between the total duration (min) of LEX/LDM phases within an LCI experiment. Values are presented as box-whisker plot. Arrows point at augmented LEX:LDM ratios seen for two individual hTCL trajectories: lower: pHEP culture enriched in cell clusters; upper: NPLC culture (close to 100% confluence). Corresponding data are given in Table 1 and Table S3. (**c**–**h**) Analysis of hTCL and KC/macrophage movement characteristics—behavioral analysis. Shown are representative *cytoethograms* (see also in the text and Figure S1) derived from LCI-based cell tracking of hTCLs in primary hepatocytes (pHEP) cultured under serum-free conditions (**c**,**d**) and NPLCs incubated in serum-supplemented (10% FBS) medium (**e**,**f**). *Cytoethograms* of Kupffer cells (KC) in NPLCs are also given for comparison (**g**,**h**). Behavioral characteristics (LDM, LEX, turns, loops) are color-coded and annotated as symbols. The corresponding LCI time-intervals are: pHEP (hTCL), (**a**) early culture (1–4 h after isolation) and (**b**) adapted culture (31–41.8 h after isolation); NPLC (hTCL and KC), (**e**,**g**) initial culture (0–3 h after passaging) and (**f**,**h**) adapted culture (6.6–15.3 h after passaging). Trajectories given in (**c**–**g**) refer to the whole monitoring period; the trajectory shown for KC/M in NPLC (**h**) covers the time-interval between 11.6 and 15.3 h, which is annotated (red lines) in (**f**). Note the series of turns in KC/NPLC (early culture) summing up to a long-distance turn.

**Figure 2:**
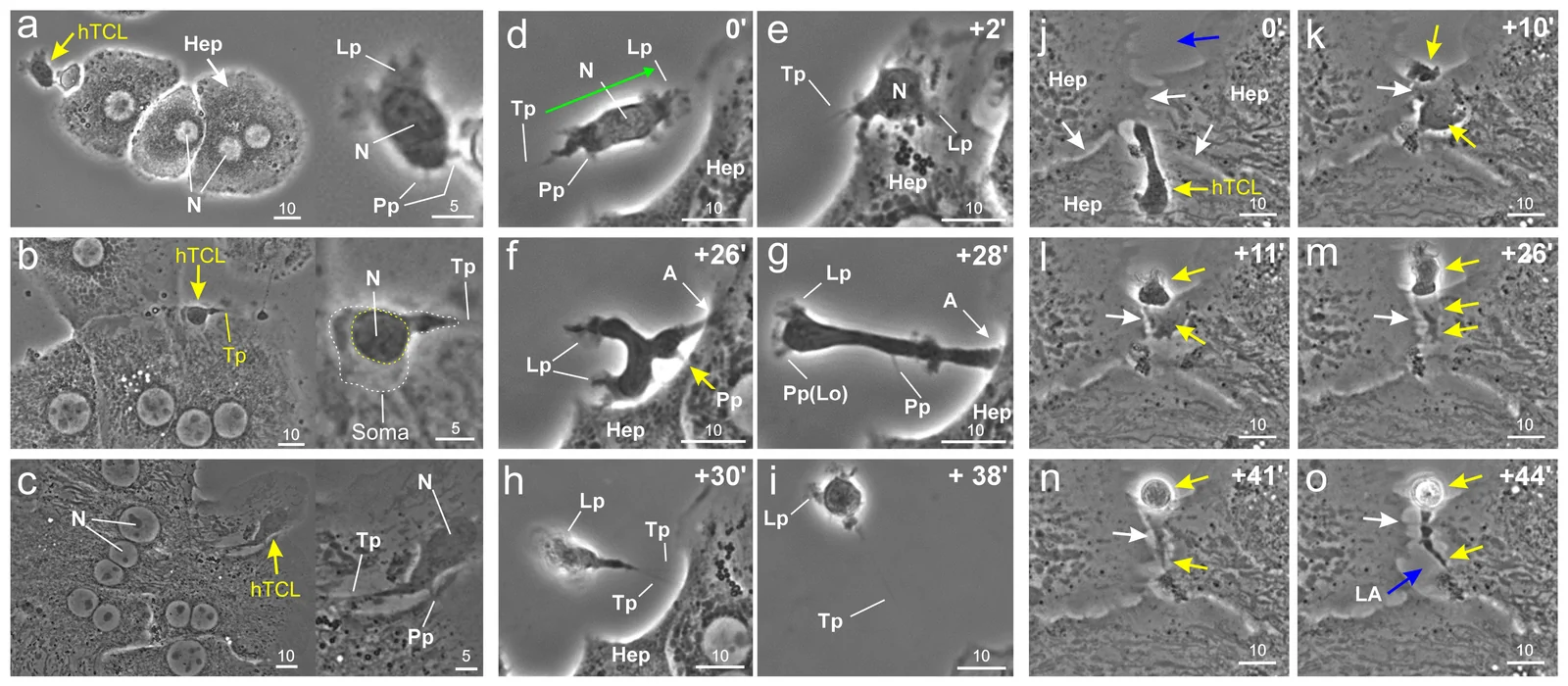
Phenotypical characteristics of hTCLs in serum-free primary hepatocyte cultures. (**a**–**c**) Cell and nuclear morphology of a hTCL monitored after 2 h (**a**), 15.4 h (**b**), and 37.4 h (**c**) of primary culture. The remarkable size differences in the cell body and nucleus (N) between the hTCL and hepatocytes (Hep) are clearly detectable, especially at the later time-points when the spread hepatocytes have developed a characteristic polyhedral shape (**b**,**c**). In the magnified view (**right panel**), the hTCL soma containing the nucleus, a lamellipodium (Lp) locating to the anterior cell pole (i.e., the movement direction), pseudopodia (Pp; in (**a**) of the filopodium type), and the extension of the soma into a telopodium (Tp) can be resolved. (**d**–**i**) hTCL plasticity over a 40 min time-interval (the sequence starts after 27.3 h of primary culture and is shown as a whole in Figure S1; see also Video S5). Upon approaching a hepatocyte (**d**), the hTCL shows an elongated phenotype with clearly detectable protrusions (Tp, Pp, Lp) oriented in the movement direction (green arrow). When the hTCL locates above the hepatocyte (**e**), it transiently develops a rounded shape (**e**) but then changes towards an elongated phenotype of remarkable plasticity with clearly detectable protrusions (**f**,**g**). (**f**,**g**) are representative of hTCLs in LEX phase developing characteristic contact sites with hepatocytes (A), in this case locating beneath the parenchymal cells. Note the contact between one pseudopodium (filopodium) and the hepatocyte surface ((**f**), yellow arrows) and another pseudopodium/lobopodium (Pp(Lo)) facing the direction of the following LDM phase (**h**,**i**). During this, the hTCL morphology is changing again towards a spindle-shaped phenotype (**h**), the hTCL transiently adopting a spherical morphology with detectable lamelli- and telopodia (**i**). (**j**–**o**) hTCL plasticity during LEX-phase-associated lumen/micro-tunnel formation between neighboring hepatocytes. The section displays three neighboring hepatocytes (Hep) with distinct cell–cell contact sites (**j**); white arrows; the blue arrow points at a cell-free area in the cell islet. Upon threading beneath the central contact site (white arrow in (**k**–**o**)), the hTCL shape changes markedly (yellow arrows), which is accompanied by a nearly complete retraction of the hepatocyte processes ((**n,o**); white arrows), generating a luminal area (LA) ((**o**); blue arrow). The sequence starts after 34.3 h of primary culture and is also shown in Video S4. Scale refers to µm.

Intriguingly, hTCLs reveal an extraordinary cell motility in primary hepatocyte and NPCL cultures. In the majority of recorded LCI video streams, individual hTCLs were detectable only for a short time-interval when quickly migrating over sections or an entire optical field. This characteristic cell migration property of hTCLs (Figure 1; Videos S1 and S2) is unique to primary liver cell cultures (parenchymal hepatocytes and mixed-NPLC cultures). In addition, hTCL migration is accompanied by a pronounced phenotypical plasticity (Figure 2; Videos S1 and S2). This differs from other, non-migrating NPLCs such as sinusoidal endothelial cells (LSECs), hepatic stellate cells (HSCs), and HSC-derived myofibroblasts, which are markedly larger, flattened cells. In contrast, Kupffer cells/macrophages (KC/M), identified in NPLC by the presence of numerous pinosomes within a dark cell soma (Video S11), display a high degree of cell plasticity and motility, which is typical of amoeboid macrophages. We have observed motile KC/M marked by quite long (≥200 µm) readily detectable process (uropodia), emerging as a thickened process from the macrophage “head-soma” with gradual thinning and local swellings (Videos S12 and S17; see Section 3.8). This contrasts with the ultra-thin, barely detectable telopodia of hTCLs, which emerge as well-demarcated, thin processes from the cell body (Figure 2a). A similar phenotype is described for telocyte telopodia, which are extremely thin processes that are close to the resolution limits of light microscopy [1]. With respect to this, however, it is noteworthy that we have also observed intermediate phenotypes of the cell body—telopodium “transition” reminiscent of that seen in amoeboid KC/M (Figure 2a–c and figure in Section 3.8). Furthermore, amoeboid KC/M move markedly slower than hTCLs, especially in NPLC established at low cell density (Figure 1; Videos S9 and S11; Table S1). Nevertheless, in dense NPLC cultures forming a cell monolayer, both KC/M as well as hTCL show a pronounced tendency to migrate below the cells of the monolayer. Moreover, in these cultures, KC/M also display behavioral characteristics as described below for hTCLs (Figure 1).

As a further characteristic, our findings show that some of the hTCLs present in rat liver are co-isolated with the parenchymal hepatocytes. This hTCL population is not separated by collagenase treatment, since they co-sediment at low speed (44× *g*) with the parenchymal cells during the washing steps and Percoll enrichment (see Section 2). This accounts for a tight attachment of hTCLs to hepatocytes, especially if hTCLs reside within non-dissociated hepatocyte aggregates (cell doublets, triplets, smaller cell clusters). Interestingly, in one hepatocyte culture examined by LCI containing an enhanced amount of non-dissociated cell aggregates, the hTCL incidence was raised to 1:45. In line with this, it has been reported recently that the strong adhesion of telocytes to neighboring cells severely hampers telocyte isolation as viable single cells [27].

### 3.2. hTCL Migration Characteristics

hTCL migration is unique among cultured primary liver cells since it is marked by exceptionally rapid movement over long distances and particular behavioral characteristics. As calculated from the recorded LCI trajectories, hTCLs move at mean velocities of 6–12 µm per min (Figure 1a) but may speed up to maximum velocities of 20–35 µm/min (highest was 46.3 µm/min) (Table S3), which is comparable to the rapid movement of activated neutrophiles under normal conditions (20–30 µm/min) [28,29]. In comparison, KC/Ms move markedly slower, with a mean velocity of about 1 µm/min and reaching top speeds of 9–13 µm/min (Figure 1a; Table S3). Interestingly, hTCL movement slows down in adapted stages of primary hepatocytes and does so less prominently in adapted NPCL cultures (Figure 1a). Figure 1a also demonstrates the opposite behavior of KC/Ms, which show an accelerated migration in adapted NPCL cultures (Figure 1a).

Of special relevance, hTCL migration displays characteristic phases of movement over longer distances (LDM, long distance migration) alternating with marked changes in movement direction (turns, loops) and phases of rather short-distance movement or local residence, suggesting some kind of exploratory behavior (LEX, local exploratory behavior). Characteristically, the mean movement velocities of hTCLs differ between LDM (8–12 µm/min) and LEX phases (4–10 µm/min) (Table 1), and the graphical presentation of hTCL trajectories focusing on ‘behavioral characteristics’—termed *cytoethogram*—without implying any ‘behavioral intentionality’ (see Figure S1), reveals further hTCL-specific characteristics (Figure 1c–f).

During the early stages of primary hepatocyte cultures, containing initially large cell-free areas, hTCLs display a pronounced exploratory behavior upon reaching individual hepatocytes or small cell groups, which is marked by an extended encircling (loops) of these cells (“green stretches” in Figure 1c; Video S1). Correspondingly, hTCLs spend markedly longer times in LEX phases, as demonstrated by an altered LEX:LDM ratio (Table 1). Since hTCLs move at higher velocity during these LEX phases, the overall distances they cover are comparable between LEX and LDM phases (Figure 1; Table 1). At later time intervals, when cell-free areas are markedly reduced due to hepatocyte flattening, hTCL behavior during LEX phases becomes more focused as hTCLs cover shorter distances during LEX phases, with markedly shortened loop periods (Figure 1d, Video S2). This is reflected by a drop in the LEX:LDM ratio (Figure 1d) and reduced contribution of LEX phases to the overall cumulative distance (Table 1). In early, freshly plated NPLC cultures, hTCLs also show pronounced LEX-phase-associated “looping” (Figure 1e, Video S11); however, their preference for LEX phases is less prominent as compared to hTCLs in early, primary hepatocytes cultures. Opposite to this, in adapted NPLC cultures, hTCLs remain markedly longer in LEX phases without displaying a distinct loop behavior (Figure 1b, Videos S9 and S10), with the LEX and LDM phases covering about the same total distance (Table 1). Like primary hepatocytes, adapted NPCL cultures are marked by progressive monolayer formation, yielding enhanced cell densities. Interestingly, in one case of confluent NPLC culture, migration of a hTCL was marked by a substantially shifted LEX:LDM ratio (Figure 1b, upper arrow), the hTCL showing only little movement within extended LEX periods. In addition, we also observed an unusually high LEX:LDM ratio for an hTCL migrating in an adapted primary hepatocyte culture with an enhanced presence of expanded cell clusters (Figure 1b, lower arrow). Taken together, these observations indicate a relationship of hTCL migratory behavior with cell density.

Finally, we did not observe LEX phases in KC/M at early NPLC culture stages when the cells showed only serial turns of their unidirectional migration (Figure 1g; Video S11). Surprisingly, however, KC/macrophage migration changed in adapted NPLC cultures, where KC/M showed similar behavioral characteristics as hTCLs, displaying distinct LEX phases, including loops and movement direction turns (Figure 1h).

### 3.3. hTCL Interaction with Neighboring Cells

During early culture stages, hTCLs appear to actively explore the surface of individual hepatocytes or small hepatocyte groups by cyclically developing protrusions and retractions of pseudopodia during LEX phases (Figure 2a, Video S1). When the hepatocytes have started to flatten substantially, hTCLs establish a distinct LDM/LEX pattern in exploring hepatocyte islets, which also includes the formation of static, transient cellular contacts at preferred attachment sites during LEX phases (Figure 1d; Video S2). Within such arrays of closely attached hepatocytes that have emerged from non-dissociated cell aggregates, hTCL migration does not follow a “random walk”. Instead, during LDM phases, hTCLs move above the cells as well as along cell–cell contacts between hepatocytes (Video S2) and frequently change their movement direction (Figure 1d). This alternates with LEX phases, where the hTCL stops moving, adheres to the hepatocyte (or growth surface), and adopts a phenotype of exceptional cell plasticity that seems to explore the hTCL surrounding (Video S2; see also Figure 2 and Video S5). hTCLs may also enter luminal areas of hepatocyte islets (Videos S2 and S4) and NPLC monolayers (Videos S9 and S10). Within such areas, hTCL behavior suggests a specific interaction with the neighboring cells (Videos S4 and S10) as outlined below. Moreover, we commonly observed hTCL movement also beneath hepatocytes and the collagen layer (Video S5) and beneath cell clusters in NPLC cultures (Video S9). In addition, we observed, in adapted NPLC cultures, that hTCLs preferentially “explored” neighboring KC/Ms, moving over the underlying adherent cells (mainly comprising LSECs and HSCs/myofibroblastoid cells) (see Video S9).

### 3.4. hTCL Exploratory Behavior Within Inter-Cell Lumina

Although seen less frequently, the behavior of hTCLs within luminal areas of hepatocyte cell islets (Video S4) and NPLC monolayers (Video S10) suggests a distinct interaction with the cell surrounding. As demonstrated in Video S4, upon reaching such an area, hTCL movement stops, and the cell enters an LEX phase, locating to the lumen formed by three neighboring hepatocytes within the cell cluster (Figure 2j). Next, the hTCL threads beneath a hepatocyte–hepatocyte contact zone (Figure 2k), part of the hTCL becoming visible at the upper, cell-free area, while the remaining cell body remains below the hepatocyte contact zone (Figure 2k–o). During this process, the protrusions of the hepatocyte cell bodies are retracting, leading to the formation of a luminal, micro-tunnel-like zone between the initially tightly attached cells (Figure 2l–o), with small cell bridges remaining between the hepatocytes (Figure 2o). The hTCL seems to explore the borders of the micro-tunnel by its pseudopodia, containing lamellipodia at their tips, which extend until the apical lamellipodium contacts a hepatocyte (Video S4). Subsequently, the hTCL section that is located beneath these hepatocyte bridges shows a vigorous motility in multiple directions before the hTCL leaves the area and enters the next LDM phase.

### 3.5. Plasticity of the hTCL Phenotype

**Figure 3:**
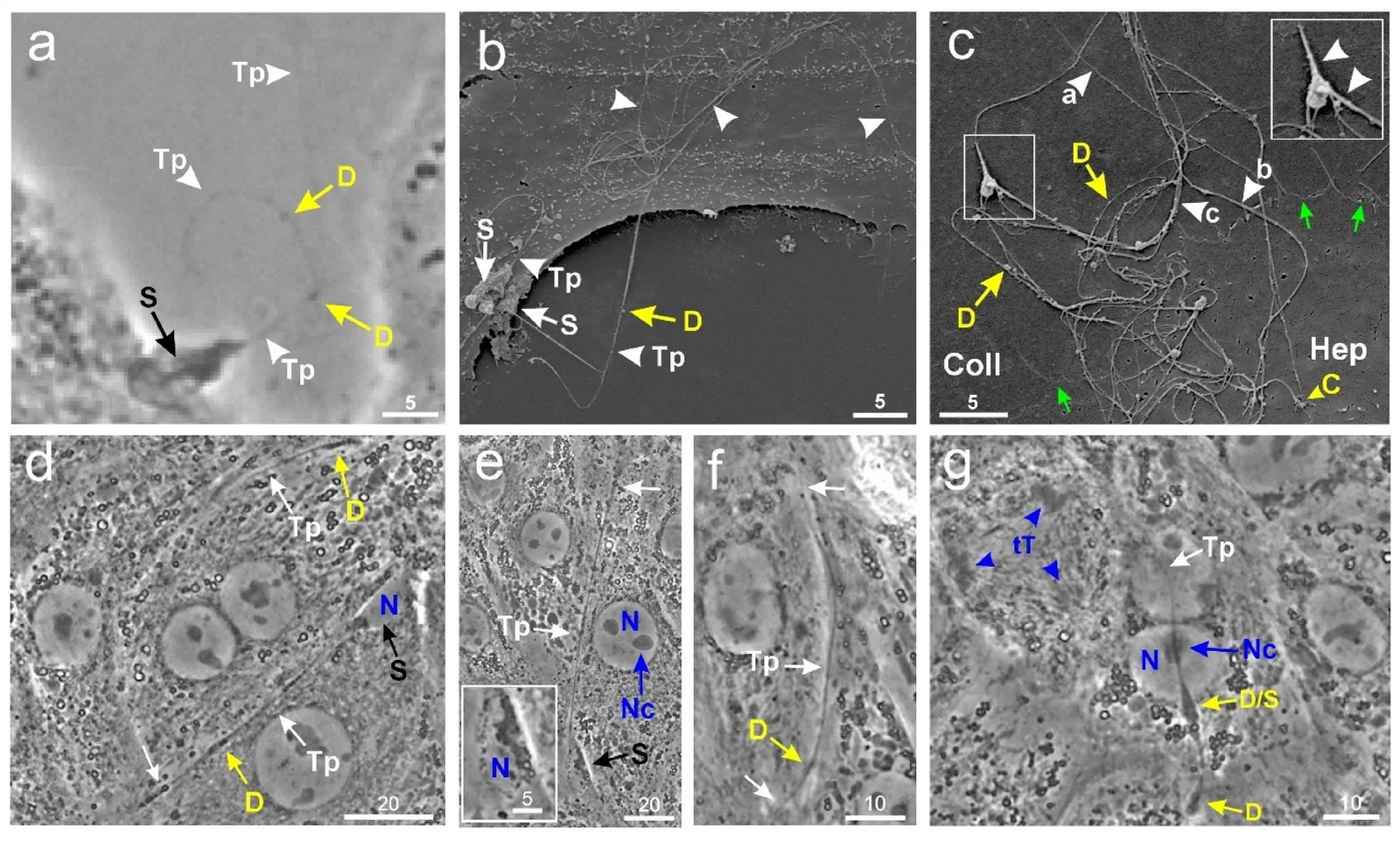
hTCL phenotype in proliferating primary hepatocyte cultures analyzed by LCI and SEM. The cultures were incubated in serum-free MEM supplemented with EGF (40 ng/mL) and insulin (0.9 µg/mL). (**a**) Phase contrast image of an hTCL locating to a hepatocyte where the soma (S) as well as the telopodium (Tp, white arrows; diameter of about 500 nm) with dilations (D; podomes) can be resolved. It is not clear whether the dark zone at the soma–Tp connection (S, black arrow) represents the nucleus (23 h 30 min after culture). (**b**,**c**) SEM inspection of hTCLs after 24 h of incubation; (**b**) two hTCLs locating to a hepatocyte membrane can be seen, one hTCL showing a very long Tp (white arrows) with several dilations (D). (**c**) Array of telopodia (white arrows) extending at a hepatocyte border (Hep; green arrows) to the collagen coat (Coll) also showing several dilations (D), Tp end-contacts to the cell surface (C), and distinct Tp-to-Tp contacts (insert). Annotated are two telopodia with diameters of 150 nm (**a**) and 286 nm (**b**) and a Tp-bundle of 430 nm diameter (**c**). (**d**–**g**) hTCL phenotypes seen in monolayers formed by proliferating hepatocytes (see Videos S6–S8). As a characteristic, hTCL visibility in proliferating hepatocytes is obscured by their small size and filamentous morphology; however, due to hTCL movement, detectability is improved by LCI. With respect to this, the reader is advised to refer to the video documentation (Videos S6–S8 and corresponding legends), which provides a detailed presentation of hTCL movement. (**d**,**e**) hTCLs that display a distinct soma (S) with a detectable nucleus (N) and telopodium (Tp) with dilations (D, (**d**)) are rarely detectable in proliferating hepatocyte cultures. The visible part of telopodium of the hTCL shown in (**d**) measures 0.7 µm in diameter and 64 µm in length, and the annotated dilation spans over 2 µm. (**e**) shows another hTCL with a less visible soma (S) also containing a nucleus (N, insert) and a long, visible Tp (107.7 µm). (**f**,**g**) Different from the hTCL phenotypes shown in (**d**,**e**), hTCLs frequently appear filamentous, marked by long, very thin telopodia (Tp) with dilations (D, (**f**,**g**)), occasionally appearing soma-like, but without detectable nucleus (**g**). hTCLs are frequently seen in the vicinity of proliferating hepatocytes marked by large nuclei (N; Nc, nucleolus; (**d**,**e**,**g**)), often during hepatocyte mitosis ((**g**); the neighboring mitotic hepatocyte displays an aberrant, tripolar telophase (tT); see also Figure 4 and Video S6). Note the size differences between the hTCL nuclei—if recognizable (N in (**d**,**e**) and insert in (**e**))—and the markedly larger hepatocyte nuclei (**d**–**g**). The time-points are 44 h 29 min (**d**), 54 h 44 min (**e**), 67 h 57 min (**f**), and 58 h 40 min (**g**) after plating in EGF/I-supplemented medium. LCI: (**a**,**d**–**g**); SEM: (**b**,**c**). Scale = µm.

hTCLs in primary hepatocyte and NPLC cultures are marked by a remarkable cell plasticity, suggesting that hTCLs continuously adapt their cell shape to the surrounding micro-environment. (Figure 2, Videos S1–S5 and S9–S11). Upon migration over longer distances, hTCLs reveal an amoeboid phenotype, marked by a rounded–piriform to elongated, spindle-shaped cell morphology shape with clearly detectable lamellipodia at the anterior cell pole (i.e., in the movement direction) and, less prominent, pseudopodia of the filopodium type at the posterior end (Figure 2a,d; Video S1). Occasionally, pseudopodia of a lobopodium type are also detectable (Figure 2g). It is sound to consider that lamellipodium-based cell motility is involved in hTCL migration (Videos S1 and S5). Interestingly, upon migration between neighboring hepatocytes, hTCLs may adopt a phenotype reminiscent of macrophages, with a detectable thickening of the posterior cell soma gradually thinning into the telopodium (Figure 2b,c; Video S2). Otherwise, due to resolution limits, telopodia are rarely visible in hTCLs and originate more sharply demarcated from the soma (Figure 2h,i and Figure 3a). As far as resolution limits allow, we found only hTCLs with either a single, very long telopodium (Figure 2i and Figure 3a,d,f) or two telopodia (Figure 2e,h).

When hTCLs are located to non-dissociated hepatocyte aggregates or within dense areas of NPLC monolayers, their cell shape transiently adopts a phenotype that sometimes is reminiscent of small KC/Ms (Figure 2b,c). Still, these hTCLs display an impressive cell plasticity and motility with respect to LDM migration and cell interactions in LEX phases (Figure 2e–g,j–o; Videos S2, S4, S5 and S9). This exceptional plasticity may allow hTCLs to continuously adapt their cell body to their migration within narrow spaces formed along/within hepatocyte contact zones (Video S2; yellow arrows) as well as beneath adherent hepatocytes (Figure S1; Video S5).

### 3.6. hTCL in EGF/Insulin-Treated, Proliferating Primary Hepatocyte Cultures

In hepatocyte cultures supplemented with EGF and insulin (EGF/I) for stimulating cell proliferation, we observed that hTCLs adopt a phenotype that was particularly different from non-EGF/I-treated cultures marked by a reduced soma containing an even smaller nucleus, which in most cases was barely or not detectable (Figure 3; Videos S6–S8). Even more characteristic, this hTCL phenotype displays at least one long telopodium with numerous dilations reminiscent of telopodium-specific podomes [1] (Figure 3a–g). Notably, in EGF-treated cultures, hTCLs were preferentially detectable within the monolayer of proliferating hepatocytes by their slightly thickened telopodia frequently locating between adjacent hepatocytes (Figure 3d–g). Inspection by scanning electron microscopy (SEM) revealed telopodia lengths of up to >100 µm at diameters between 150 to about 300 nm with several dilations (Figure 3c), which is consistent with telocyte telopodia [1,3,4]. In addition, we also observed hTCLs bearing larger telopodia bundles of >400 nm diameter (Figure 3c). Thus, the filamentous hTCL structures seen in EGF/I-treated hepatocyte cultures by LCI-phase contrast (Figure 3a,d–g; Videos S6–S8) may either represent thickened telopodium sections or telopodia bundles. SEM analysis further revealed contact zones between hTCLs and the hepatocyte surface at the telopodium-terminus (Figure 3b) and telopodium–telopodium junctions (Figure 3c). With advanced cultivation in the presence of EGF/I (>48 h), the identification of hTCLs within the dense monolayers by LCI phase contrast became even more difficult. In these cultures, hTCL were visible only transiently as filamentous bodies, resembling thickened telopodia (Figure 3c), occasionally with intervening soma-like dilations (Figure 3g), but without any detectable nucleus (Figure 3f,g; Video S8). Although the hTCL incidence appeared higher upon prolonged EGF/I treatment of hepatocytes, this observation was not substantiated due to the difficulties in detecting hTCLs at advanced timepoints, as mentioned.

**Figure 4:**
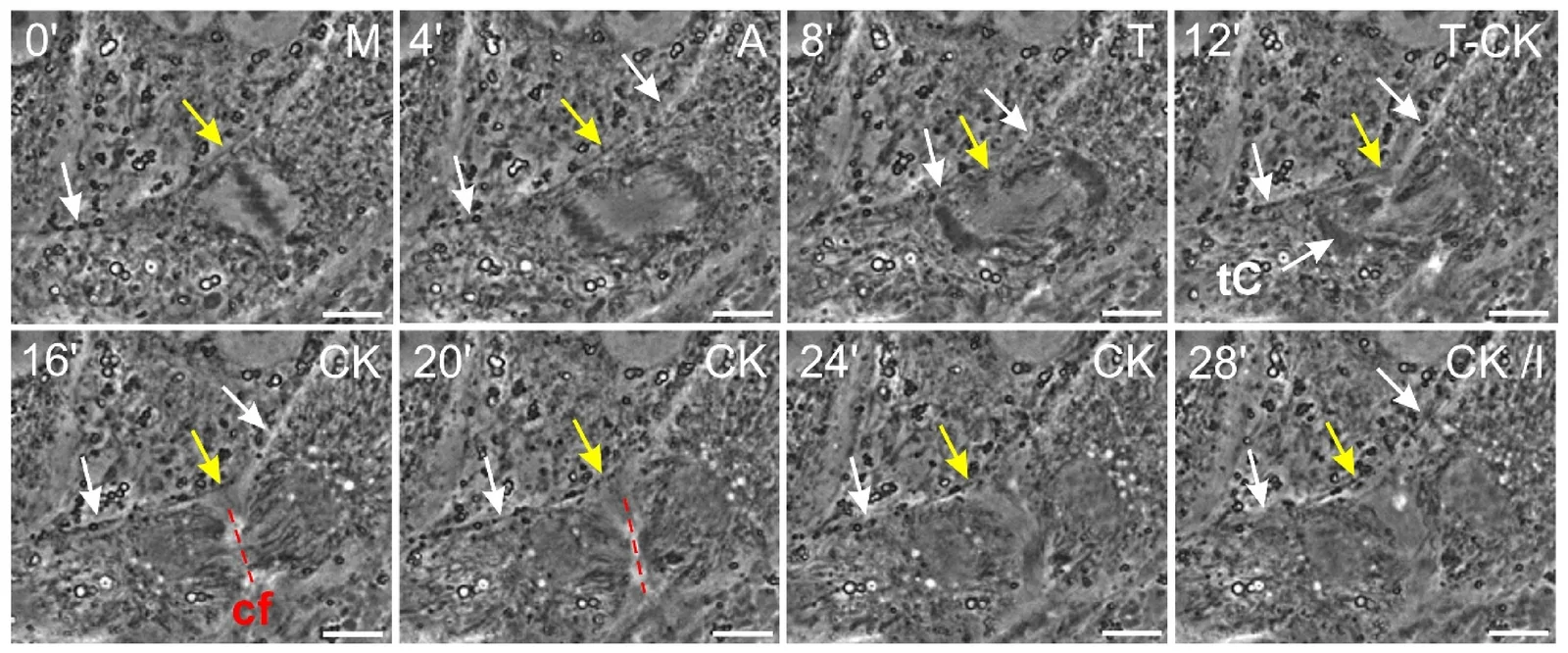
hTCL and hepatocyte mitosis. The hTCL/telopodium (white and yellow arrows) stays in close contact with a neighboring mitotic hepatocyte proceeding through mitotic meta- (M) and anaphase (A). During telophase (T) and cytokinesis (CK; CK/I—cytokinesis/interphase), the shape of the telopodium follows movement of the dividing hepatocyte (yellow arrow) at the cleavage furrow (cf, red dotted line). The sequence is taken from Video S6 and starts after 46 h 30 min of primary culture under proliferative stimulation. As commented in Figure 3, the reader is advised to refer to the documentation of Video S6 (see also Supplement: legends to Videos S1–S17) for improved traceability of the shown context. Scale = 10 µm.

Paralleling the morphological changes, hTCL behavior showed special characteristics in proliferating hepatocyte cultures. As documented in Videos S6–S8 and shown in Figure 4, hTCLs showed a distinct association with dividing hepatocytes. Preceding the onset of a mitotic event, hTCLs frequently located close to neighboring hepatocytes, which showed prominent rotation of the nucleus and entered mitotic prophase after nuclear orientation had stabilized (Videos S6–S8). During subsequent mitosis, the hTCL telopodium stayed in tight contact with the hepatocyte cell membrane close to the mitotic figures, following the movement of the dividing cell/nucleus during telophase and cytokinesis (Figure 4; Video S6). This ‘telopodia dragging’ towards the hepatocyte cell surface during cytokinesis (Figure 4) is frequently seen (Videos S6–S8). Interestingly, we also observed on a regular basis that hTCL telopodia orientated parallel to the mitotic spindle axis (i.e., perpendicular to the mitotic equatorial plane) during metaphase and perpendicular to the cleavage furrow (Figure 4; Videos S6 and S7). The dragging of the telopodium into the cleavage furrow may indicate a physical connection between telopodia and dividing hepatocytes in EGF/I-treated cultures.

### 3.7. Nuclear Size of hTCLs

Similar to telocytes, hTCLs contain very small nuclei with detectable heterochromatin accumulation at the nuclear lamina. In non-EGF/I-stimulated hepatocyte cultures, the size differences between the nuclei of hTCLs and hepatocytes are readily detectable during early culture and become even more impressive in adapted cultures when the hepatocytes have flattened (Table S1; Figure S3). Referring to area measurements, hepatocyte nuclei are about 3-4-fold larger than that of hTCLs. Of relevance, the mean area given in Table S1 for hepatocyte nuclei refers mainly to di- and tetraploid nuclei and only a low number of octoploid nuclei as usually seen in primary rat hepatocyte cultures [30,31]. Nevertheless, considering hTCLs as diploid cells, these measurements account for a high degree of “chromatin compaction” in hTCLs. Moreover, hTCL nuclei also display a marked plasticity accompanying the morphological changes in the cell body (Figure 2a–e), and the hTCL nucleus is not always resolvable, which applies especially to hTCLs in EGF/I-stimulated hepatocyte cultures (Figure 3d–g).

### 3.8. hTCL Versus Macrophages (KC/M)

**Figure 5:**
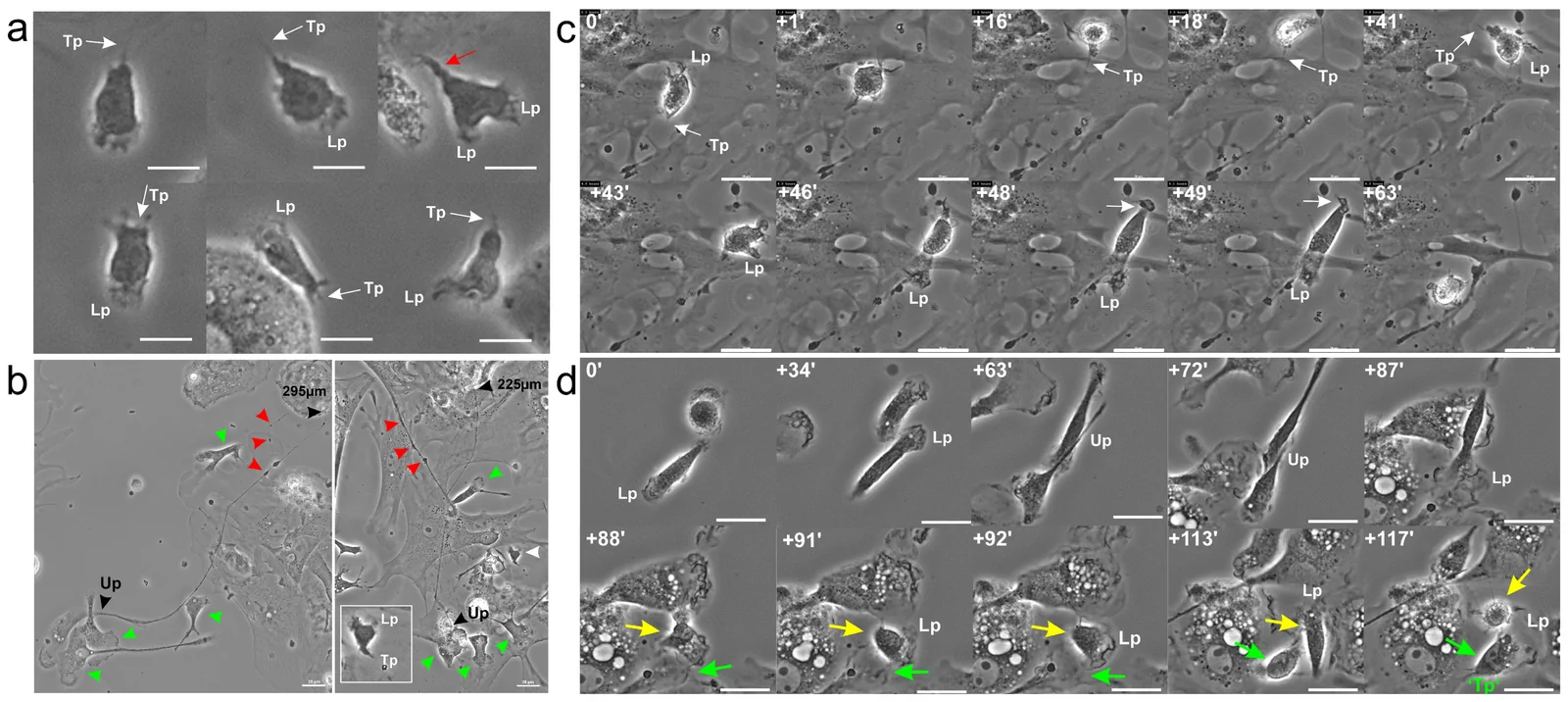
hTCLs and KC/Ms: phenotypical differences and intermediate phenotpyes (**a**). Characteristic phenotypes of moving hTCLs in early primary hepatocyte cultures. Lamellipodia (Lp) are readily detectable at the anterior cell pole, pointing into the movement direction. The “tailing” telopodia (Tp) are barely resolvable. In some hTCL phenotypes, the soma shows a thickened transition zone to the telopodium (see also Figure 2b,c,h). Scale = 10 µm. (**b**) KC/Ms (green arrows) of different sizes and morphologies in NPLC cultures. Some of the KC/Ms display very long (>200 µm) processes, uropodia (Up; black arrows) which show pronounced dilations (red arrows) reminiscent of telocyte/telopode podoms. In the (**right panel**) of (**b**), an hTCL is detectable (white arrow) with a branched lamellipodium and a hardly resolvable telopodium (Tp) (insert). Notably, even the hTCL soma is not easily detectable in this field. (**c**,**d**) hTCL-KC/M transitional morphologies: (**c**) shows an hTCL in a confluent NPLC culture. The hCTL adopts a macrophage-like morphology during a LEX phase between 48 and 63 min. LCI has shown that the morphology of this cell does not revert to a hTCL phenotype (as shown in (**a**)). Instead, this cell continues to display this rounded/compact shape alternating with the macrophage-like morphology. (**d**) shows a smaller KC/M at early NPLC culture (immediately after plating), which develops into a larger cell of typical, amoeboid-macrophage morphology (0–87 min). Within one min (87–88), the KC/M (yellow arrow) shrinks markedly and displays an hTCL phenotype (91–117 min), subsequently disappearing below the left, neighboring cell. Another KC/M (green arrow), appearing at +88 (min) shows a similar phenotype-transition between +113 and +117; notably, as can be seen at +117 (min), this cell develops an intermediate morphology with a pronounced, macrophage-like lamellipodium (Lp, green) in the anterior and a well-demarcated process at the posterior end of the soma, highly reminiscent of telopodia (‘Tp’, green). Sequences are from Videos S13 and S14. Scale = 20 µm.

Due to their size and migration characteristics, hTCLs are well distinguishable from other cell types in NPLC cultures (Videos S9 and S12). Nevertheless, in high-density monolayers of NPLC cultures, we observed hTCLs that displayed a macrophage-like phenotype (Figure 5, Video S13), which was also seen occasionally in hTCLs moving along cell-clusters in hepatocyte cultures (Figure 2b; Video S2). This suggests a tendency of hTCLs to adopt a macrophage-like phenotype in the presence of increasing cell densities. Vice versa, in NPLC cultures, we observed KC/Ms that developed an intermediate phenotype, showing morphologies reminiscent of both hTCLs and KC/Ms (Figure 5; Video S14). As a further phenotypical variant, also seen in NPLC cultures, we detected small, rounded cells reminiscent of both hTCLs and KC/Ms, which showed little or no movement (Video S15). Hence, the presence of hTCLs to small KC/M intermediates in the liver cannot be ruled out. This could also be indicated by the presence of small, macrophage/hTCL-like cells in NPLC cultures that transiently developed a rounded, tuberous phenotype comparable to hTCLs in LEX phase (see Videos S3 and S10), followed by migration to the basal side of the underlying cells, where they developed a phenotype characteristic of KC/Ms (Video S16).

Finally, related to the difference between hTCLs and KC/Ms, we find it appropriate to report a further observation we made frequently in NPLC cultures: large, slowly migrating amoeboid KC/M, which develop extremely long, thickened processes with lengths of >200 µm, bearing podome-like dilations as seen in telocyte-telopodia but easily distinguishable from these by light microscopy (Figure 5, Video S17).

## 4. Discussion

We describe hepatic cells characterized by a small cell body (<12 µm), a nucleus-to-cell ratio of 0.3 (i.e., 30% of the cell is covered by the nucleus), and, most specifically, the presence of very thin (<0.2 µm diameter) cell processes, which in most cases cannot be resolved upon light microscopical observation. Accordingly, these hepatic cells match the ‘hallmarks of telocyte identification’ defined by Popescu and co-workers [1,3,4]. Consequently, we named these cells hepatic telocyte-like cells, the add-on term ‘-like’ referring to pending hTCL ‘immunotyping’, which will be discussed below.

Our main findings, that hTCLs reveal pronounced migration-based exploratory behavior and an exceptional cell plasticity, have to our knowledge not been reported for any other hepatic cell type. Although we have investigated hTCLs in female rats only, we assume that these findings are not restricted to one sex only. Concerning telocytes isolated from other tissue, LCI data are sparse and do not provide detailed information on cell motility or migratory behavior [32,33]. In contrast, it has been documented that telocytes mediate the migration of keratinocytes, dermal microvascular endothelial cells, and mesenchymal stem cells [34,35]. hTCL migration reaches mean velocity maxima of 20–35 µm/min with a measured top speed of 46.3 µm/min. This is comparable to the rapid movement of activated neutrophiles (20–30 µm/min) under normal growth conditions [28,29]. Concomitantly, hTCL migration is marked by distinct movement characteristics, the foremost being exploration of their cellular environment. Upon such cell–cell contacts, hTCLs slow down and finally reside either on top or beneath the neighboring cells as well as within lumina/cavernas formed between cells inside cell groups. During such LEX phases, hTCLs apparently explore their surroundings, as indicted by impressive changes in cell morphology. During LEX phases, hTCLs may transiently establish distinct cell–cell contacts to stabilize themselves while ‘exploring’ nearby cells. In addition, between LEX phases, hTCLs move quickly along or between cell contacts during LDM phases connected with a subsequent LEX phase. Following the same rationale as above, this could indicate an active scouting process of hTCLs for distinct receptor–ligand interactions. With the combination of a small, highly ductile cell body and very thin telopodia, hTCLs are well adapted to serve spatial restrictions, like loose cell–cell contacts, as seen in cultured hepatocyte clusters or in the Space of Disse in the liver. However, additional factors may also play a role in hTCL movement. For instance, telocytes isolated from liver para-cancer tissue express matrix metalloproteinase 9 (MMP-9) [36], as do telocytes of catfish (*Clarias gariepinus*) and quail embryos, which may support movement of cells such as macrophages in the course of development [37,38].

The small size and plasticity of the cell soma may represent a prerequisite for hTCL movement through narrow spaces, which may also apply to telocytes. In this context, a small nuclear volume is advantageous. This is visualized by the massive size differences we have observed between hepatocyte and hTCL nuclei. Even more critically, in the dense monolayers of EGF-treated hepatocyte cultures, the soma and nuclei of hTCLs are barely visible or virtually undetectable. It is sound to consider that this comes along with a further reduction in the nuclear volume. If so, chromatin condensation will define the lower size limit for hTCL nuclei, which can only be underrun by a reduction in ploidy. Aneuploidy, including the presence of haploid chromosome sets, has been documented in mesenchymal cells [39,40] and malignancies related to mesenchymal origin (for review see [41]). In the 1950s, haploid metaphase chromosome sets were also reported in rat liver [42,43], which was not further investigated. Interestingly, in an investigation on rat spleen telocytes, Chang and co-workers describe a telocyte in mitotic division, where the two daughter cells, still connected by a cell bridge during advanced cytokinesis, vary markedly in size and DNA content, the smaller cell showing only minute amounts of labeled DNA [32]. In light of this, the existence of haploid telocytes cannot be excluded.

The association of hTCL telopodia with dividing hepatocytes in EGF/I-treated hepatocyte cultures cannot be explained adequately at present. Whether this indicates a “passive” effect of “telopode dragging” of hTCLs locating close to hepatocytes during cytokinesis or accounts for a specific mechanism of hTCL–hepatocyte interaction in mitotic spindle orientation remains the subject of further investigation employing functional studies. Nevertheless, mitotic spindle orientation is crucial to tissue formation [44,45]. This may apply to the compensatory growth of hepatocytes. In addition, cardiac tissue renewal also involves telocyte-based interstitial networks for stem cells and precursors within the stem cell niche [46,47], which hypothetically may also require adequate organization of mitotic spindle mechanics.

As stated above, by morphological criteria, hTCLs match the definition of telocytes and thus may be considered as a hepatic telocyte subtype. Indeed, several tissue-specific telocyte subtypes have been described, which share structural similarities (as hTCLs do) but significantly differ in their immunohistochemical characterization [4,48]. Hence, the exact nature of hTCLs needs to be addressed by further immunologic examination, which is pending at present. Of relevance to this, Popescu and Faussone-Pellegrini reviewed the evidence existing in 2010 for telocyte-specific markers with restraint, conceding differences among tissues and animal species and the limitation of using markers common to cells of mesenchymal origin such as CD34 and c-kit in ICC and ICLC [1]. Still today, a unique, selective telocyte marker has not been found, and a set of a markers comprising CD34, c-Kit/CD117, and PDGFRα/β is commonly used for telocyte identification. Among them, CD34 and PDGFRα are considered most reliable for identifying telocytes, including those of the liver [4]. Nevertheless, in his excellent review, Vannucchi states that telocyte characterization by these markers may be interfered with by particular physiologic contexts. Recent findings point in a similar direction. In late 2025, Erkan and co-workers reported the presence of telocyte-like cells (TLCs) in the umbilical vein stroma [49]. Similar to our investigation, the authors have defined TLCs by structural and phenotypical similarities with telocytes. In addition, umbilical-vein TLCs were positively identified as telocytes by immunoreactivity for vimentin, c-Kit, and PDGFRα but were negative for CD34. As a detail relevant for immuno-typing of telocytes in vitro, the authors state that immunoreactivity for c-Kit and PDGFRα was strong in tissue sections but weak in cultured umbilical TLCs. In addition, by use of other markers, umbilical TLCs were also identified as multipotent stromal cells, and the authors concede that despite the identification of these cells as TLCs and multipotent stromal cells, none of the employed methods allowed a definitive characterization.

In liver tissue sections, telocytes were identified based on the combined positive double-immunofluorescence for marker-pairs such as CD34/vimentin, CD34/PDGFRα/β, and CD34/c-kit [9,10,16], and recently in primary liver telocytes too [22]. Intending to follow a similar approach using NPLC cultures due to their higher hTCL incidence, however, we faced potential concern with respect to marker specificity since other cells contained in NPLCs also show immunoreactivity for telocyte markers. For instance, besides CD34+ LSECs (liver sinusoidal endothelial cells), CD34+ endothelial progenitor cells [50,51,52], CD34+ hepatic fibroblasts/myofibroblasts [53], and CD34+/CD32+ Kupffer cells/Kupffer cell precursors have been reported, which also express c-kit/CD117 [54]. Moreover, the adult liver contains hematopoietic stem and progenitor cell classes, which will also “stain positive” for CD34 and c-kit [55,56]. This also holds true for hepatic CD34+/c-kit+ cells, considered to represent biliary epithelial cell precursors [57]. Finally, immunoreactivity for PDGFRα/β may be seen in hepatic pericytes, periductal fibroblasts, and potentially also hepatic stellate cells and Kupffer cells/macrophages [53,58,59,60,61,62,63]. In addition, the similarities we have observed between hTCLs and KC/Ms may interfere with hTCL immuno-typing. It cannot be excluded at this time that hTCLs adopting a macrophage phenotype—and vice versa, small KC/macrophages adopting hTCL morphology—may cross-react with these telocyte as well as macrophage markers, a scenario that is reminiscent of the complication faced by Erkan and co-workers in umbilical telocytes/multipotent stromal cells, as outlined above. Moreover, the presence of hTCL–macrophage intermediate phenotypes could indicate that at least some of the hTCLs represent a macrophage-related telocyte subset. In this context, we may also refer to the high motility and behavioral characteristics of hTCLs, which clearly distinguish hTCLs from Kupffer cells/macrophages (Figure 1). However, this does not hold true for the slower moving or even resting (LEX phase) hTCL–macrophage intermediates. Hence, the characterization of hTCLs on the immunological level demands an extended approach that allows an unambiguous discrimination between hTCLs, telocytes, and macrophages.

In addition, the scarcity of hTCLs and ‘impurity’ of NPLC cultures is a further factor of considerable relevance. Screening the existing literature on rat telocytes in PubMed identified a limited number of in vitro investigations using primary cultures enriched in telocytes. Common to the published ‘standard protocols’ for telocytes isolation are the (i) enzymatic digestion (collagenase; trypsin) of explanted, minced small-sized tissue blocks; (ii) low-speed centrifugation; and (iii) “sieving” the collected cell suspension via 100 µm and/or 40 µm nylon mesh and culturing the cells in serum-supplemented (10% FBS) DMEM. In several cases, the initially isolated cells are plated and incubated for a short time period, and only the non-adherent cells then are used as primary telocytes, growing as adherent cells that need to be trypsinized for subculture. It appears noteworthy that telocytes grown in primary telocyte cultures established by this procedure are markedly larger and display readily detectable, thicker telopodia, which are rather reminiscent of the Kupffer cells/macrophages we have seen in NPLC cultures (Figure 5; Videos S11 and S17). This is in conflict with the original definition of telopodia, which are very thin and therefore “*are anyway below the resolving power (0.2 µm) of light microscopy*” [1]. Notably, in preparing NPLC cultures, we followed a similar protocol. Starting with the two-step collagenase liver in situ perfusion, the collected, raw-cell suspension was filtered via a 120 µm nylon mesh, and the fraction of parenchymal hepatocytes sedimented by ultra-low-speed centrifugation (44× *g*; 10 min). From the supernatant, the entire NPLC fraction then was collected by low-speed centrifugation (240× *g*; 10 min), and the cells were cultured in DMEM/10% FCS. Different from the standard protocols, we washed the cells after an initial culture period of 2 h but used the adherent cells (and not the ‘washed fraction’) for further culture. Besides several different cell types (e.g., HSCs, LSECs, fibroblastoid cells), NPLC cultures obtained by this procedures contained hTCLs (at an incidence of 1:29) and Kupffer cells/macrophages at markedly higher abundance.

It is evident that more specific protocols need to be elaborated for preparing highly enriched cultures of liver telocytes/hTCLs, and probably primary telocytes from other tissues too, with minimum contamination by other cell types, foremost KC/Ms. Separation methods such as density gradient centrifugation may improve purity, although it remains to be demonstrated whether telocytes can be quantitatively separated from macrophages by this methods. An alternative, promising approach has been reported by Romano and co-workers employing a two-step immunomagnetic microbead-assisted isolation procedure [64]. Nevertheless, strong physical interactions of telocytes with neighboring cells as described by Canella et al. [27], which may also underly the co-isolation of hTCLs with primary hepatocytes, may hamper such an approach, as well as other, FACS-based isolation/enrichment methods.

Finally, hTCLs/telocytes and macrophages may not share only phenotypical similarities but could also be connected functionally. It has been demonstrated that telocytes may form stromal synapses with neighboring cells, including macrophages [7,17]. In the liver, KCs normally locate to the liver sinusoids. However, they may transmigrate into the Space of Disses, where they can interact with HSCs and hepatocytes [65,66]. Hence, telocyte–KC/macrophage interactions such as described for the gut may also exist in the liver. Interestingly, the findings of Yang at al. [22] indicate a pluripotency of liver telocytes that can differentiate into adipocytes, osteoblast, and cardiocytes. This pluripotency depends on the activity of NPC1, a gene product that is essential to the export of cholesterol out of lysosomes. Depletion of the NPC1 gene causes the intralysosomal accumulation of lipids, including of oxysterols, which pathologically manifests as Nieman–Pick syndrome, a characteristic lysosomal-storage disorder [67]. Intriguingly, NPC1 is mainly expressed in hepatic macrophages (KCs), where mRNA levels are substantially augmented upon liver injury, including oxidant intoxication (CCl_4_) [68]. Moreover, this report has also demonstrated that knock-out of myeloid NPC1 stimulates liver inflammation and fibrosis in mouse models and Npc1-defiency promotes activation of macrophages with M1 and proinflammatory polarization. Together, this accounts for a hepatoprotective role of NPC1, mediated via myeloid/macrophage activities. With respect to this, it is tempting to speculate on an NPC1-mediated differentiation of pluripotent telocytes into cells of myeloid origin, including macrophages and Kupffer cells, in response to liver injury. The finding of hTCL–macrophage intermediates could account for a similar process occurring in primary hepatocytes and NPLC cultures. Notably, cells of both culture types are exposed to pro-oxidant conditions during cell isolation (liver perfusion) and passaging (NPLC subculture), as well as mild hyperoxia upon cell culture, contexts that could hypothetically promote hTCL differentiation into macrophages.

Under stress conditions, hTCLs/telocytes and macrophages could share additional functional properties, especially with respect to PDGFR-based signaling. In neurologic contexts (brain ischemia, lumbar spinal stenosis), PDGFR activation has been shown to mediate macrophage migration/infiltration to the wounded tissue [59,60]. Following the concept of mild oxidative stress acting on cultured primary hepatocytes and NPLCs, conditioned media of both may contain PDGF, which is known to be secreted by KCs, activated HSCs, as well as hepatocytes under stress conditions [69,70]. From this, it is tempting to speculate on a PDGFR-stimulated, NPC1-controlled differentiation of hTCLs/telocytes into migratory macrophage intermediates, which serve the surveillance of liver tissue integrity under conditions of oxidative stress. It will be the subject of future investigation to elucidate the exact nature of hTCLs and hTCL/telocyte–macrophage intermediates and their potential physiological relevance in the liver, as well as tissue contexts beyond.

In conclusion, we have identified liver-derived telocyte-like cells that display a distinct migratory behavior and may adopt structural similarities to liver macrophages. Further investigations has to define the exact immunological signature of these cells and the relation between hTCLs/telocytes and macrophages under the physiologic aspects.

## Figures and Tables

**Table 1:** Analysis of hTCL trajectories.

Culture Type ^a,b^			Velocity (µm/min)		Total Phase Distance (% of the Cumulative Distance) ^c^		LEX:LDM ^c^ Time Ratio ^d^
			LDM	LEX	LDM	LEX	
hTCL	pHEP	Early [8]	12.6 ± 1.9	9.9 ± 4.0	54.4 ± 27.6	54.6 ± 27.6	1.74 ± 1.54
		Adapted [6]	11.4 ± 2.3	5.7 ± 2.8	68.9 ± 20.6	31.1 ± 20.6	1.28 ± 1.32
	NPLC	Early [3]	8.5 ± 3.1	6.1 ± 2.5	68.2 ± 19.3	31.8 ± 19.3	0.79 ± 0.71
		Adapted [8]	8.5 ± 1.8	4.6 ± 1.5	49.1 ± 16.2	50.9 ± 16.2	2.95 ± 3.46
KC/M	NPLC	Early [3]	2.9 ± 1.5	0	-	-	-
		Adapted [3]	2.9 ± 0.5	2.4 ± 0.1	68.7 ± 2.0	31.3 ± 2.0	0.56 ± 0.18

^a^ pHEP, parenchymal hepatocytes; NPLC, non-parenchymal liver cells; KC/M, Kupffer cells/macrophages. ^b^ LCI-monitoring periods: pHEP: early (0–24 h after plating) and adapted culture (24–48 h after plating); NPLC: early (0–3 h after plating) and adapted culture (>6 h after plating). *N* = number of different monitored hTCL trajectories from *N* = 4 (pHEP) and *N* = 2 (NPLC) independent primary cultures, comprising the velocity measurements of a total (LDM + LEX) of 2343 and 3177 frames for hTCL trajectories of 8 and 6 hTCL in early and adapted hepatocyte cultures, respectively. In NPLC cultures, hTCL movement velocities were determined in 421 and 2681 frames (LDM + LEX) for 3 and 8 hTCLs in early and adapted cultures, respectively. For KC/M, three representative trajectories were selected with a total number of velocity measurements of 410 (early) and 655 (adapted) frames. See Table S3. The migration differences between hTCL and KC/M are also displayed Videos S11 and S12. ^c^ Cumulative distances are given in Table S3. ^d^ The LEX:LDM ratio is the ratio between the total duration of LEX/LDM phases of a trajectory within the whole LCI-monitoring time-period.

## Data Availability

The original contributions presented in this study are included in the article/Supplementary Materials. Further inquiries can be directed to the corresponding author. All data supporting the findings of this study are available within the paper and its Supplementary Materials. All materials are available on reasonable request from the corresponding author.

## Supplementary Materials

The following supporting information can be downloaded at: https://www.mdpi.com/article/10.3390/cells15161476/s1, Figure S1: Analysis of hTCL behavior summarized as *cytoethogram*; Figure S2: hTCL plasticity; Figure S3: Cell and nuclear size of hTCL in primary hepatocytes. Table S1: Cell scores; Table S2: Morphometric analysis of hepatocytes and hTCLs; Table S3: hTCL trajectory analysis–Overview. Video S1: hTCL movement during initial hepatocyte culture; Video S2: Behavioral characteristics of hTCLs in adapted hepatocyte cultures; Video S3: Tracking of hTCL behavior; Video S4: hTCL plasticity I. Interaction with hepatocytes; Video S5: hTCL plasticity II; Video S6: hTCLs in EGF/insulin-treated primary hepatocyte cultures I; Video S7: hTCLs in EGF/insulin-treated primary hepatocyte cultures II; Video S8: hTCLs in EGF/insulin-treated primary hepatocyte cultures III; Video S9: hTCLs in mixed cultures of primary non-parenchymal hepatocytes (NPLC); Video S10: hTCL LEX behavior in a luminal area within a dense NPLC culture; Video S11: hTCL and KC movement in NPLC I; Video S12: hTCL and KC movement in NPLC II; Video S13: hTCL–KC/M intermediate phenotypes I; Video S14: hTCL–KC/M intermediate phenotypes II; Video S15: hTCL-KC/M intermediate phenotypes III; Video S16: hTCL–KC/M intermediate phenotypes IV. Video S17: KC/M phenotypes/uropodia. Videos are shown in https://www.ebi.ac.uk/biostudies/studies/S-BSST3395, accessed on 10 August 2026.

## Abbreviations

ECM	Extracellular matrix
EGF	Epidermal growth factor
EGF/I	Combined treatment with EGF and insulin
hTCL	Hepatic telocyte-like cells
HGF	Hepatocyte growth factor
LCI	Live-cell imaging
LDM	Long distance movement
LEX	Local exploratory behavior
LSEC	Liver sinusoidal endothelial cells
MEM	Minimum essential medium
NPC1	C1 Niemann–Pick disease Npc1 gene
KC/M	Kupffer cells/macrophages
PDGFR	Platelet-derived growth factor receptor
SEM	Scanning electron microscopy
TGFα	Transforming growth factor α
TGFβ1	Transforming growth factor β1
Tp	Telopode/telopodium
