# Supplementary material for: Live-Cell Imaging of Telocyte-like Cells in Primary Liver Cell Cultures
Source: Cells. 2026 Aug 18;15(16):1476. doi: 10.3390/cells15161476 (PMC13511627; doi:10.3390/cells15161476)
Supplement: Supplementary file 1 [file cells-15-01476-s001.zip › cells-4240965 Legends to Video S1-S17.pdf]

Legends to Videos S1-S17.

Video S1. hTCL movement during initial hepatocyte culture. Shown is a group of primary hepatocytes and a markedly smaller, fast moving hTCL between 1 to 2.4 hours of primary culture. The hTCL displays an ameboid phenotype featured by characteristic protrusions (lamellipodia, pseudopodia) and pronounced cell migration. Suggestive of an exploratory behavior, the hTCL trajectory shows phases of rapid movement over cell-free areas that alternate with phases of encircling the attached hepatocytes. Note the marked flattening of the hepatocytes and the marked size difference between the hTCL and the hepatocytes and hepatocyte nuclei (mono- and binucleated cells), at the end of the video sequence. The timecode [0.0 hours] refers to 1 hour after plating the freshly isolated cells.

Video S2. Behavioral characteristics of hTCLs in adapted hepatocyte cultures. The video sequence starts after 24 hours [Timecode = 0.0 hours] of primary culture and monitors hTCL movement at restricted to a group of non-dissociated hepatocytes. Initially, the hTCL shows a rounded cell shape (0.1h; TC, white arrow) and develops the characteristic ameboid phenotype upon migrating above the hepatocytes. Similar to the hTCL shown in Video S1, hTCL migration is characterized by alternating phases of long-distance movement (LDM) and local exploratory behavior (LEX). For instance, this is seen after 3.4-3.5 hours, when the hTCL changes from an LDM to an extended LEX phase marked by a characteristic local interaction between the hTCL and the neighboring hepatocytes (LEX E, red arrow; see also Video S4). Of note, during LDM phases, the hTCL adopts a macrophage – like phenotype with a detectable telopodium (reminiscent of the uropodia of macrophages) upon migration between adjacent hepatocyte borders (yellow arrows at 7.0 and 8.0 hours; white arrows pointing at the hTCL).

Video S3. Tracking of hTCL behavior. The video displays the hTCL trajectory shown in Video S2 highlighting LDM phases (color-coded) within the hepatocyte array and LEX phases (red) mainly seen at the array borders. Within LDM phases, loops and turns can also be seen. Analysis of this hTCL trajectory is representative for morphometric analysis of hTCL migratory behavior carried out in this study (see also Figure S1).

Video S4. hTCL plasticity I. Interaction with hepatocytes. The video represents a magnified view of the LEX phase E seen in Video S2, highlighting hTCL activity at a contact zone between two neighboring hepatocytes (white arrow). Initially, the hTCL (yellow arrow) migrates beneath the hepatocyte contact zone which is accompanied by an almost complete separation of the hepatocytes with few remaining cell bridges (white arrow at 0.6 hours) and formation of a luminal space (white arrow at 0.8 hours). Subsequently (timepoints > 1.4 hours), the marked movement of the hTCL pseudopodia at the hepatocyte cell borders (white arrow) suggests an intensive exploratory behavior of the hTCL.

Video S5. hTCL plasticity II. The video gives another representative example of hTCL-phenotype plasticity accompanying the alternating LDM and LEX phases. LCI monitoring started after 46h51min [Timecode = 0 hours] of serum-free primary culture. The hTCL displays a typical amoeboid shape when migrating over a cell-free area (e.g. 0 – 0.2 hours) and an elongated macrophage-like phenotype upon movement in vicinity to the hepatocytes (e.g. 0.2 – 0.6 hours). Between 0.3 – 0.5 hours, the hTCL migrates beneath the hepatocytes. Note that at 0.4 hours, the hTCL is out of focus as the focal plane was adjusted to the hepatocyte surface locating above. When moving away from the hepatocyte array (0.7 hours), the hTCL again shows a characteristic hTCL ameboid cell shape, displaying a lamellipodium at its anterior front (movement direction) and two telopodia becoming faintly detectable at the posterior pole of the cell soma.

Video S6. hTCLs in EGF/insulin-treated primary hepatocyte cultures I. The LCI-video starts after 41h45min [Timecode = 0.5 hours] of cultivating the cells in EGF/I [40ng / mL] and insulin [0.9 µg / mL] supplemented, serum-free MEM. Initially barely resolvable within the hepatocyte monolayer, the hTCL becomes detectable more clearly at 1 hour (white arrows), showing a filamentous phenotype with a defined cell soma (S) and an extended telopodium (the left arrow is pointing at a telopodium dilation /

podome). At later time-points (e.g. 2.7h) the hTCL nucleus becomes detectable in the soma. As frequently seen in EGF/I-treated primary hepatocyte cultures, the hTCL is located between hepatocyte-hepatocyte contact sites, close to a large (presumably tetraploid) hepatocyte nucleus which starts rotating at 2.8 hours (yellow arrow pointing at a large nucleolus). At 3.4 hours, the hTCL develops marked dilations, locating close to the still rotating nucleus (similar observations are displayed in Video S7 and S8). After rotating at about 180°, the rotation stops and the nucleus enters mitotic prophase with chromosomes appearing (red arrow). At this time-point, the hTCL with its telopodium becomes tightly located to the hepatocyte membrane aside the prophase nucleus where it remains until the end of the mitotic process. At metaphase, the equatorial plane of the mitotic spindle, as defined by the orientation of the metaphase chromosomes stays perpendicular to the telopodium (5.2 hours, red and white arrows). During subsequent ana- and telophase, and in particular in the course of cytokinesis, the hTCL/telopodium follows the mitotic movements and becomes drawn towards the forming cleavage furrow (5.3 – 5.5 hours). Upon completion of cell division, the hTCL/Telopodium still stays closely located to the daughter cells.

Video S7. hTCLs in EGF/insulin-treated primary hepatocyte cultures II. This LCI-sequence monitors three hTCLs (white, blue and green arrows) between 50.0 – 65.3 hours [Timecode = 0 hours] of serum-free primary hepatocyte culture in the presence of EGF and insulin. All hTCLs show a pronounced filamentous phenotype. At an initial time-interval (1.8 hours), the hTCL to the left (white arrows) displays a soma (S) and a nucleus (lower white arrow). In contrast, no soma and nucleus are detectable in the other hTCLs which can be identified only by their moderately thickened telopodia (blue arrows). Like the hTCL seen in Video S6, both hTCLs locate close to adjacent hepatocyte nuclei, which rotate (yellow arrows) preceding mitotic division (red arrow). At 2.3 hours, the telopodium of the upper hTCL (blue arrows) is also oriented perpendicular to the hepatocyte metaphase equatorial plane and terminal cleavage furrow (red arrow) and remains tightly attached to the hepatocyte membrane close to the nuclear zone during hepatocyte mitosis. The hepatocyte, located to the left hTCL (white arrows) shows three phases of nuclear rotation (between 1.4 – 2.4, 4.0 – 5.2 and 7.7 – 11.4 hours). Preceding the third rotation phase, this hTCL/telopode also becomes tightly located to the hepatocyte membrane aside the hepatocyte nucleus (6.8 hours, 2nd upper white arrow) the telopodium subsequently developing a dilation (brown arrow, 8.9 hours) during the third rotation phase of the hepatocyte nucleus. Immediately before hepatocyte mitosis starts at 11.7 hours (red arow), the hTCL/telopode migrates above the nucleus and moves out of focus (10.7 hours; white arrows). The telopodium is visible again at late metaphase and anaphase (12.1-12.2 hours; white arrows) when it orients perpendicular in the z-plane to the cleavage furrow of the yet divided hepatocytes locating beneath (time > 12.6 hours; white and brown arrow). At this time-interval (12.4. hours), a third hTCL becomes detectable showing a soma (lower green arrow) and a telopodium with dilations (upper green arrows) which stretches alongside the same hepatocyte-to-hepatocyte contact zone.

Video S8. hTCLs in EGF/insulin-treated primary hepatocyte cultures III. Completing the presentation of hTCLs, this video sequence (also starting after 50 hours [Timecode = 0.0 hours] of serum-free primary hepatocyte culture in the presence of EGF and insulin) displays the complex hepatocyte – hTCL relationship seen in proliferating hepatocyte monolayers. Three hTCLs(1-3) are followed: hTCL(1) and hTCL(2) are detectable by their telopodes (0.0 hours, white and blue arrows) at the beginning of the video sequence. hTCL(3), also of the filamentous phenotype, becomes visible by its telopode 9.4 hours later (green arrows). All three hTCLs/telopodes show dilations, in hTCL(1) one dilation locates to the neighboring hepatocyte membrane close to nucleus area (0.0 hours; brown arrow). The behavior of all three hTCLs follows that explained in Video S6 and 7, showing an association with rotations of the hepatocyte nuclei, a tight attachment to the hepatocytes during advanced mitosis and a perpendicular orientation with the cleavage furrow during cytokinesis (6.8. hours, hTCL(2) – blue arrow; 13.9 hours hTCL(3) green arrow; orientation is less apparent in hTCL(1) at 3.8 – 4.2 hours, brown arrow). Of special interest, hTCL(1) develops prominent telopodium dilations during the whole monitoring period (white arrows), but the nucleus cannot be resolved, even in the frame at 4.6 hours, when the dilation becomes

most prominent (2nd upper white arrow) and the telopode extends over a very long distance (white arrows). Mitosis of the hepatocyte to the upper left is preceded by nuclear rotation (7.1 hours; yellow arrow), the telopode of hTCL(1) locating unusually distant from the hepatocyte / nucleus area (upper white arrow); however, during the following, tripolar mitosis of the adjacent hepatocyte, the telopode locates close to the to the dividing hepatocyte during cytokinesis, also orienting perpendicular to the cleavage furrow forming between two of the three daughter nuclei (8.8. hours; upper white arrow). Shortly later (9.4 hours), the same telopode locates above the neighboring binucleated cell, both nuclei entering mitosis. At metaphase, the telopode located above the mitotic hepatocyte is oriented perpendicular to the mitotic equatorial plane (9.9 hours; upper white arrows) and also follows telophase movement and the cleavage furrow upon cytokinesis (10.1 – 10.4 hours). Finally, hTCL(3) appears at 9.6 hours in vicinity to a binucleated hepatocyte prior to mitosis (yellow arrow). At 11.2 hours, a dilation of the telopodium of hTCL(3) is detectable that again is oriented perpendicular to the mitotic equatorial plane of the adjacent hepatocyte metaphase nucleus and subsequent cleavage furrow (12.2 hours; green arrow). Terminally, the telopodium of hTCL(3) in manner similar to the other hTCLSs, accompanies hepatocyte mitosis displaying nuclear rotation (10.6 hours; yellow arrow to the right section) before entering mitosis at 12.5 hours, completing cell division at 14.2 hours (the green arrow points at a telopode dilation formed at the location of the mitotic cleavage furrow).

Video S9. hTCLs in mixed cultures of primary non-parenchymal hepatocytes (NPLC). The LCI-video starts 6.6 hours after passaging primary NPCs (passage 2), plated at high cell density. At this time-point the NPLC culture has developed a sub-confluent monolayer of enlarged, flattened cells. With respect to cell morphology, the monolayer mainly consists of LSECs. The large cell locating to the right lower sector, featured by a large nucleus, enlarged pinosomes (vacuoles) and a lighter, inhomogeneous areal within the cell body likely represents a HSC, possibly in the process of adopting a myofibroblastoid phenotype). Smaller Kupffer cells/macrophages (KC), marked by a dense, cell body with elongated processes showing a prominent plasticity and very slow cell movement are preferentially locating to the top (as well as between) the cell monolayer. Initially (yellow arrow), a hTCL with pronounced LEX behavior can be seen within a cell lumen reminiscent of the hTCL/hepatocyte interaction shown in Video S4. Note the KCs (red arrow) in proximity to this area. After an extended time-period (9.3 hours; yellow arrow), the hTCLs moves away from the areal and locates beneath a non-spread cell aggregate before continuing with a further LDM phase (9.7 hours; yellow arrow). The hTCLs exits the section and re-enters the frame at 10.1 hours (yellow arrow) with continue LDM/LEX phases until end of the sequence, the hTCL preferentially locating close to KCs during LEX phases. Note the prominent differences in hTCL and KC movement which can be followed, especially after the hTCL has re-entered the section.

Video S10. hTCL LEX behavior in a luminal area within a dense NPLC culture. Shown is the magnified view from Video S9, of a hTCL (yellow arrow) location to a luminal area within the NPLC monolayer. The LEX phase extends over > 3 hours (6.6 – 9.3 hours), KCs (red arrow) also entering the filed. Note the macrophage-like phenotype the hTCL adopts at the end of the sequence marked by a thickened process of the posterior cell soma (yellow arrow at 9.4 hours).

Video S11. hTCL and KC movement in NPLC I. The LCI-video starts one hour after plating primary NPLCs upon the 1st passage at low cell density. The movement of three hTCLs (yellow arrows to the left section side) and three Kupffer cells /macrophages (KC/M) (red arrows to the right section) can be seen. Note the KC/M residing on the surface of markedly larger NPLC. Characteristic of macrophages, the KC/Ms display numerous small vacuoles representing pinosomes (light puncta within a dark cell soma). Similar to Video S9, the hTCLs moving within this area show a pronounced tendency of encircling the resident KC/Ms. Following the hTCL migration is complicated by the high velocities as compared to slowly moving large, amoeboid KC/Ms with long, tailing uropodia.

Video S12. hTCL and KC movement in NPLC II. Like Video S12, the section shows a NPLC culture upon the 1st passage when monolayer formation is progressing. The Video impressively demonstrates the size differences between hTCLs (yellow arrow) and KC/Ms (red arrows) which display very long processes (uropodia) of > 200 µm length (see Figure 5). Also in this example the hTCL preferentially encircles / contacts that resident KC/Ms in the lower cel array.

Video S13. hTCL-KC/M intermediate phenotypes I. The Video shows a hTCL in a NPLC culture of increased cell density at LDM-LEX phase transition. Between 4.0 to 4.3 hours, the hTCL adopts a phenotype reminiscent of small KC/Ms. See also Figure 5.

Video S14. hTCL-KC/M intermediate phenotypes II. Shown is a LCI-stream acquired in low density primary NPLC cultures 1 hour after plating (1st passage). In the first video section, movement of a small KC/M (yellow arrow) towards a group of non-flattened NPLCs is seen. Upon reaching the cell patch (after 1.27 hours (yellow arrow), the KC/M more or less instantly compacts displaying a morphology, reminiscent of hTCLs. At the end of the sequence, this hTCL-macrophage intermediate cell adopts a highly condensed, "tuberous" cell shape and terminally moves beneath the cell cluster to the left. After 1.33 hours another KC/M (green arrow) appears at the lower left section. Initially, this KC/M displays a well-defined macrophage phenotype with a large lamellipodium. At 1.82 hours, the cell body of this KC/M also rapidly compacts and transiently adopts a hTCL-macrophage intermediate phenotype similar to the other KC/M.

Video S15. hTCL-KC/M intermediate phenotypes III. The video sequence displays two very small, condensed, rounded cells in a low-density NPLC culture 1 hour after plating (1st passage). Both cells develop a hTCL phenotype (yellow, green arrows), without significant movement. Only at the end of the sequence both cells start to move away from their position. Upon reaching the border of the cell to the upper right, one of the cells (yellow arrow) develops a condensed and rounded shape, similar to the hTCL-like cell in Video S14 (yellow). Similar changes of the phenotype are frequently seen in hTCLs during LEX phase in primary hepatocytes (Video S2,S4) and NPLC cultures (Video S9,10). Remarkably, a small KC/M which at the beginning of the sequence locates between the two rounded cells moves to the neighboring cells to the upper left shows a comparable behavior.

Video S16. hTCL-KC/M intermediate phenotypes IV. The LCI-stream shows another section of the NPLC cultures monitored in Video S15. Similar to the small KC/M shown in Video S15, two of the three small KC/M s develop a condensed, rounded "tuberous" shape reminiscent of hTCLs upon moving over the cell surface of the large, neighboring cell. Following the KC/M to the right (white arrow), part of the cell body of this KC/M – hTCL intermediate cell becomes detectable, moving out-of-focus, beneath the underlying cell (red arrow), showing a macrophage morphology.

Video S17. KC/M phenotypes / uropodia. This LCI-sequence shows KC/Ms move across section of a low-density NPLC culture. Most noticeable is the slowly moving KC/M in the center marked by a large cell soma of high plasticity and a well-resolvable, long, branched process (uropodium) displaying several dilations. Note that the uropodium with > 200µm length covers almost the whole diagonal of the optical field (Scale = 20µm).
