# Supplementary material for: Live-Cell Imaging of Telocyte-like Cells in Primary Liver Cell Cultures
Source: Cells. 2026 Aug 18;15(16):1476. doi: 10.3390/cells15161476 (PMC13511627; doi:10.3390/cells15161476)
Supplement: Supplementary file 1 [file cells-15-01476-s001.zip › cells-4240965 Supplemental Figure S1.pdf]

Figure S1. Analysis of hTC behavior summarized as *cytoethogram*

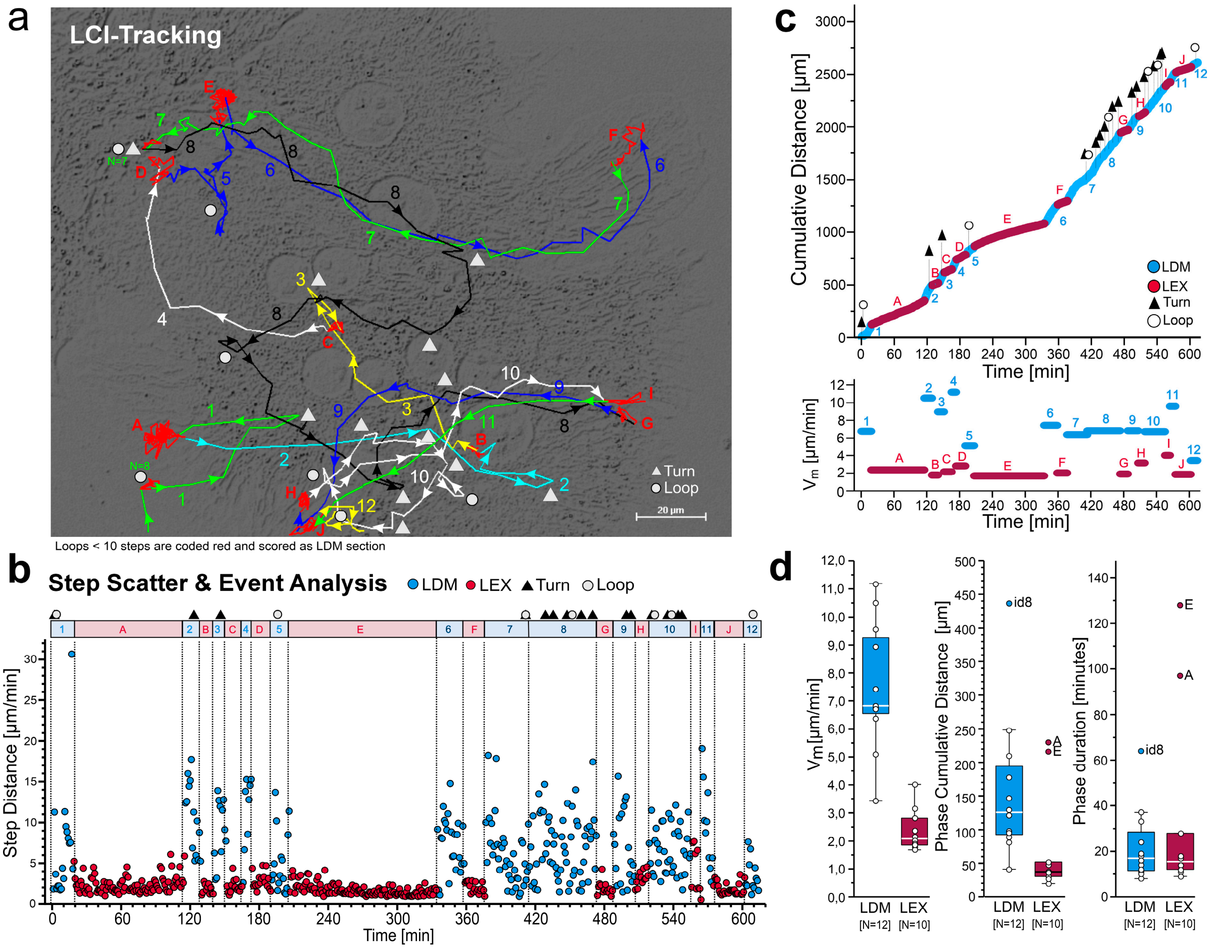

Figure S1. Analysis of hTCL behavior summarized as *cytoethogram*. (**a)** manual tracking (one-minute time intervals) of a representative hTCL trajectory recorded in a primary culture of parenchymal hepatocytes between 31- 41.8 hours (see Video S3). (**b)** scatter analysis of the tracking data. Each dot represents the distance covered by the hTC within one minute. LDM and LEX phases are color coded; turns of movement direction and movement loops (i.e. movement turns comprising 360 degrees) are annotated above the plot. Local short-distance movements covering less than 10 minutes are shown in red in Figure 1a but are assigned to the corresponding LDM. (**c)** hTCL-tracking based presentation of hTCL behavior as *cytoethogram*. We use the term with reference to the description of behavioral characteristics of cell migration, which does not imply any "behavioral intentionality". The total distance covered by hTCL movement within the monitored 10.8 hours is displayed with color-coded annotation of behavioral characteristics (LDM, LEX phases, turns, loops). The diagram below displays the mean velocity V_m [µm/minute] of hTCL movement within each individual LDM and LEX phase. (**d)** Box-whisker plots summarizing the differences between LDM and LEX phases for V_m,(left), the cumulative distance [in µm] covered within each phase (mid) and the duration [in minutes] of each individual movement phase (right). The number of LDM and LEX phases analyzed is given in parentheses.
