# Supplementary material for: Live-Cell Imaging of Telocyte-like Cells in Primary Liver Cell Cultures
Source: Cells. 2026 Aug 18;15(16):1476. doi: 10.3390/cells15161476 (PMC13511627; doi:10.3390/cells15161476)
Supplement: Supplementary file 1 [file cells-15-01476-s001.zip › cells-4240965 Supplemental Figure S2.pdf]

Figure S2 hTCL plasticity

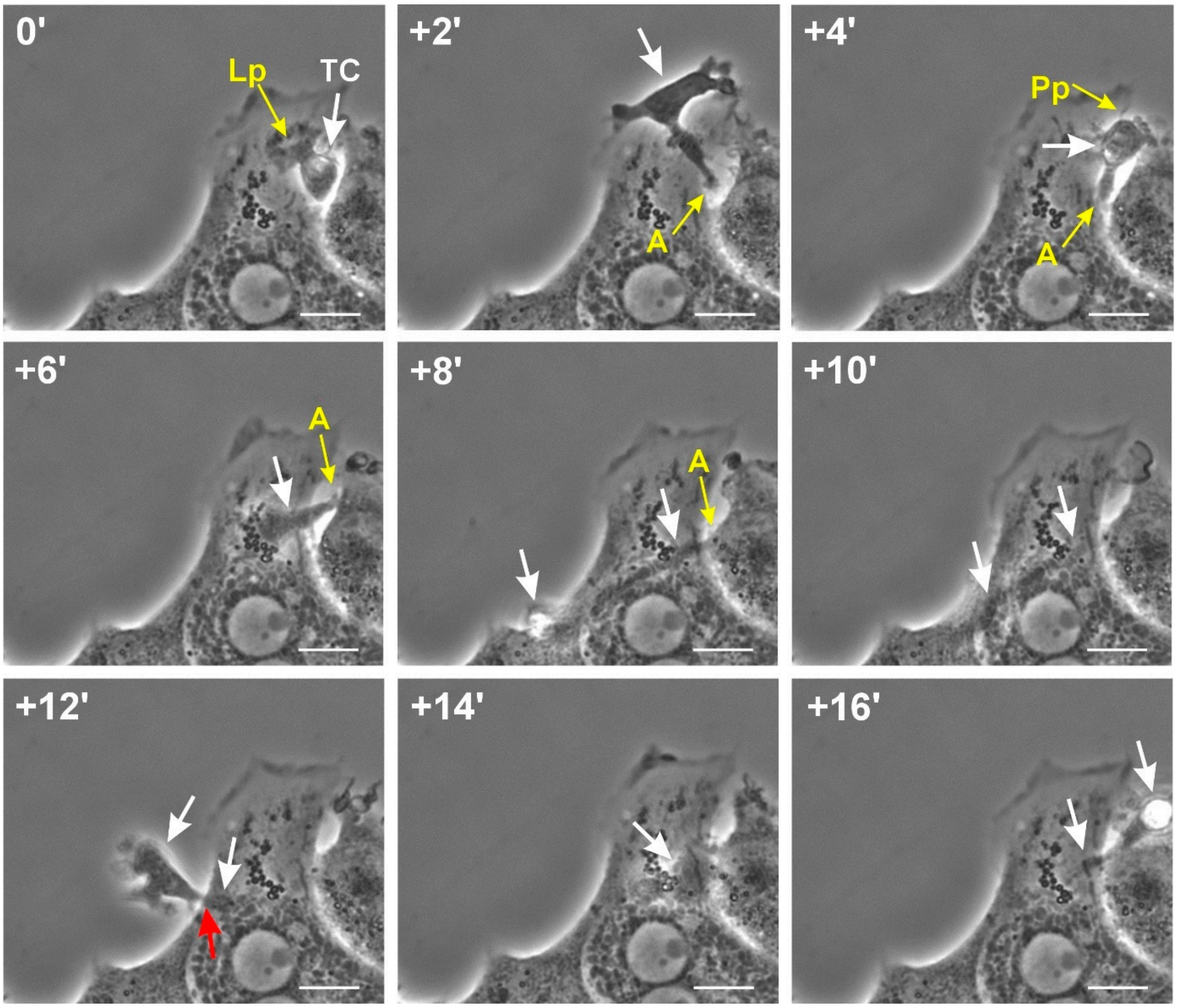

Figure S2. hTCL plasticity. Hepatocytes cultured in serum-free MEM were monitored by LCI after 46h39min of primary culture [= 0′; indicated time are in minutes; the image series is taken from LCI-Video 5]. The hTCL displays a rapid series of remarkable morphological changes. Within the first two minutes, the initial (0′) rounded cell with a clearly detectable lamellipodium develops an ameboid-mesenchymal phenotype and as typically seen for hTCL in LEX phase, is attached to a small cell lumen formed between the two neighboring hepatocytes (A). After a further two minutes, the hTCL resumes a rounded cell shape with detectable pseudopodia but remains anchored to the luminal substratum (Pp) (+4′). Subsequent to this, still attached to the substratum (A), the hTCL stretches beneath the hepatocyte located to the left (+6′ - +10′), the cell body appearing as a "blurred structure" (white arrows). Finally, the hTCL appears to the left of the hepatocyte (+12′; white arow). Note that the hTCL is still out of focus (white arrows) indicating its anchorage to the growth surface below the hepatocyte, which focused above (red arrow). Thereafter, the hTCL migrates back to its former location again below the hepatocyte (+14′ - +16′; white arrows). The further sequence can be followed in Video S5. Demonstrating its pronounced exploratory behavior, after end of the sequence shown here, the hTCL continues to migrate beneath the hepatocyte and after +20′ returns to the location where it was detectable at +12′.
