# Supplementary material for: Live-Cell Imaging of Telocyte-like Cells in Primary Liver Cell Cultures
Source: Cells. 2026 Aug 18;15(16):1476. doi: 10.3390/cells15161476 (PMC13511627; doi:10.3390/cells15161476)
Supplement: Supplementary file 1 [file cells-15-01476-s001.zip › cells-4240965 Supplemental Figure S3.pdf]

Figure S3 Cell and nuclear size of hTCL in primary hepatocytes

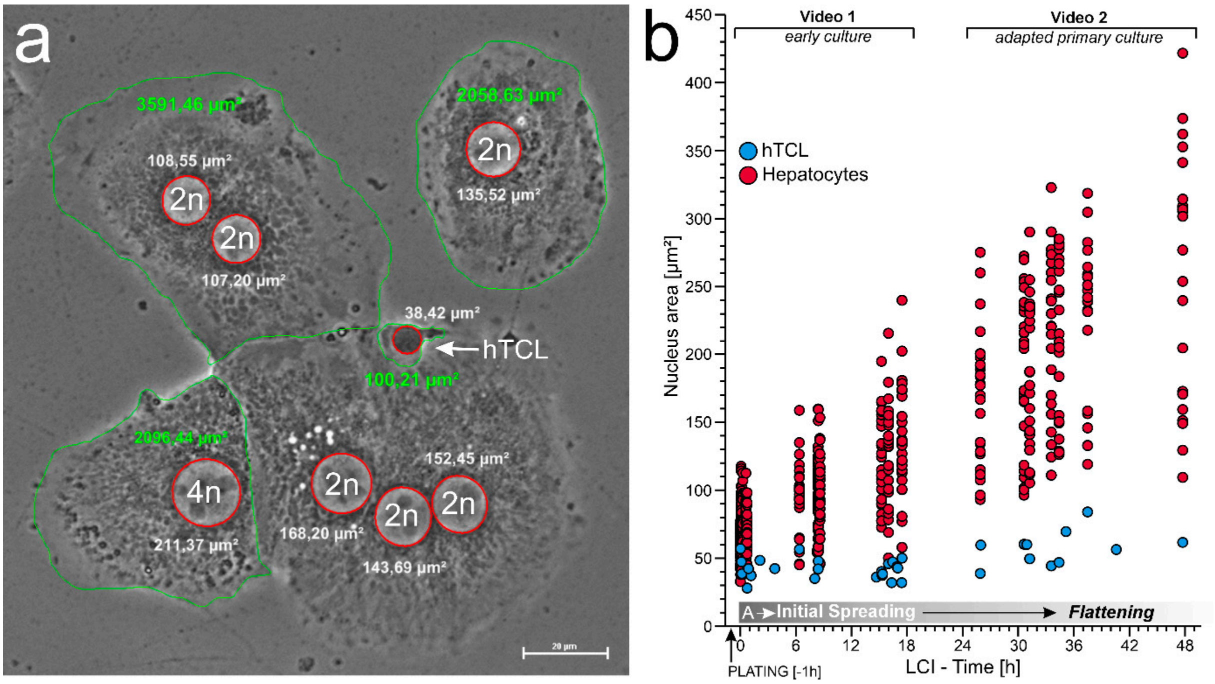

Figure S3. Cell and nuclear size of hTCL in primary hepatocytes. The cultures were established in serum-free MEM and monitored by LCI over 48 hours of culture. Nuclear size [µm²] was determined in selected LCI-images at different timepoints. (**a**) Shown is a hTCL located to a group of flattened hepatocytes after 15.4 hours of culture (see also Figure 2b). The image is representative of hTCLs in primary hepatocyte cultures at earlier time-points, when the hepatocytes adopt a well-spread, polyhedral shape. Cell and nuclear sizes are given by green and white numbers, respectively. Green lines demarcate the cell borders which are not clearly detectable between the hepatocytes within the cell group (a mono- and a binucleated hepatocytes) to the lower right. Besides the prominent difference in cellular and nuclear size, the hTCL differs from the neighboring hepatocytes also with respect to the nucleus-to-cell ratio (by area): hTCL = 0,38 compared to 0,06-0,1 for the hepatocytes (see also Supplemental Table I). (**b**) Morphometric analysis of nuclear size – changes for two different hTCLs in primary hepatocyte cultures. Two LCI-experiments were performed monitoring the cells during an early culture phase (Video 1, plating (0h) to 18h; hTCL: N=1, hepatocytes: N=25 (16 mono- / 9 binucleated cells) with a total of 34 nuclei) and an adapted culture (Video 2, 24 – 48hours; hTCL: N=1, hepatocytes: N= 23 (18 mono- / 5 binucleated cells) with a total of 28 nuclei). After initial cell attachment (A), the initially rounded hepatocytes start spreading (see also Video S1) and continue flattening on the growth substratum during further cultivation. As shown in the scatter plot, this is paralleled by a continuous expansion of the cell nuclei (red dots). Nuclei in hTCLs show no comparable size changes, their size expanding moderately at continued incubation (Video 2; adapted culture) which is also seen in. The prominent homogeneity of hepatocyte nuclei reflects the presence of di-, tri- and tetraploid hepatocytes reported for primary hepatocytes cultures [1,2].

References

1. Eckl PM, Strom SC, Michalopoulos G, Jirtle RL. Induction of sister chromatid exchanges in cultured adult rat hepatocytes by directly and indirectly acting mutagens/carcinogens. Carcinogenesis. 1987;8(8):1077-83.

2. Eckl PM, Bresgen N. The cultured primary hepatocyte and its application in toxicology. J Appl Biomed. 2003;1:117-26.
