# Supplementary material for: Live-Cell Imaging of Telocyte-like Cells in Primary Liver Cell Cultures
Source: Cells. 2026 Aug 18;15(16):1476. doi: 10.3390/cells15161476 (PMC13511627; doi:10.3390/cells15161476)
Supplement: Supplementary file 1 [file cells-15-01476-s001.zip › cells-4240965 Table S1.pdf]

Table S1. Cell scores

Cell culture [1]	Fields [2]	Cells [3]	Cell density [4]	hTCL ratio [5]	
				hTCL / cells	hTCL / field
pHEP [N=15]	193 (43)	5105 (54)	25,07 ± 7,69	0,0081 ± 0,0072 (1:123,5)	0,216 ± 0,242
NPLC [N=4]	34 (20)	1226 (54)	36,77 ± 15,47	0,0342 ± 0,0392 * (1:29,3)	1,312 ± 1,463 *

[1] pHep, primary parenchymal hepatocytes. NPLC, primary non-parenchymal liver cells. The numbers of independent experiments (i.e. primary cultures prepared from different animals) are given in parentheses. [2] Values refer to the total number of individual optical fields that were scored; the number of fields where hTCLs were detectable is given in parentheses. [3] Values refer to the total number of scored cells; the total number of detected hTCLs is given in parentheses. [4] Cells per optical field [mean ± SD].[5] number of hTCLs related to the total number of cells and fields [mean ± SD]. * p=0,026 (hTCL ratio cells) and p=0,03 (hTCL ratio fields) compared to pHEP (Two-tailed Mann Whitney U-test; exact significance). Correlations: Spearman's rho for cell density versus hTCL ratio (cells) = 0,083 (pHEP) and p=0,769 (NPLC; p=0,2).
