# Supplementary material for: Live-Cell Imaging of Telocyte-like Cells in Primary Liver Cell Cultures
Source: Cells. 2026 Aug 18;15(16):1476. doi: 10.3390/cells15161476 (PMC13511627; doi:10.3390/cells15161476)
Supplement: Supplementary file 1 [file cells-15-01476-s001.zip › cells-4240965 Table S2.pdf]

Table S2. Morphometric analysis of hepatocytes and hTCLs

Cell			Nucleus	N:C ratio [2]
Type	Diameter [μm] [1]	Area [μm²]	Area [μm²] [1]	
pHEP [a]	38,15 ± 4,52	1184,38 ± 275,24	96,66 ± 23,62	0,089 ± 0,015
hTCL [b]	10,88 ± 0,44	93,98 ± 7,72	28,51 ± 1,53	0,306 ± 0,033

pHEP, parenchymal hepatocytes. Measurements were performed in LCI-streams of early cultures. Cell and nucleus measurements were acquired for pHEP in [a] N=5 selected optical frames of 5 independent LCI-experiments between 10 and 18 hours of culture comprising a total score of 73 mononucleated hepatocytes and [b] N=4 different hTCL trajectories acquired over the whole time-period between 1-18 hours of primary culture; measurements were performed only in optical frames where hTCL nuclei could be clearly detected comprising a total score of 219 individual hTCL measurements. Values are mean ± SD. [1] The mean nuclear area comprises mainly di- and tetraploid and a low number of octoploid nuclei (see also Figure S3).

[1] Equivalent diameter (EqD): $EqD = \sqrt{\frac{4\,x\,Cell\,Area}{\pi}}$. [2] N:C ratio= Nucleus area / Cell area.
