# Supplementary material for: Live-Cell Imaging of Telocyte-like Cells in Primary Liver Cell Cultures
Source: Cells. 2026 Aug 18;15(16):1476. doi: 10.3390/cells15161476 (PMC13511627; doi:10.3390/cells15161476)
Supplement: Supplementary file 1 [file cells-15-01476-s001.zip › cells-4240965 Table S3.pdf]

Table S3. hTCL trajectory analysis – Overview

Culture type [a,b]			Frames [1]	Time [2] [min]	Cumulative Distance [3] [µm]	Velocity - Vm [µm/minute]			
						Mean Vm (overall)	Vm min [4]	Vm max [4]	Vm max /body area [5]
hTCL	pHEP	Early [8]	2343	60 – 735	836,3 – 7379,8	11,2 ± 2,4	1,1 ± 0,7 (0,53)	29,6 ± 4,1 (35,97)	2,7 ± 0,4
		Adapted [6]	3177	381 – 735	2611,5 – 7351,1	8,6 ± 2,3	0,7 ± 0,2 (0,41)	35,4 ± 7,2 (46,30)	3,3 ± 0,7
	NPLC	Early [3]	421	75 –180	500,2 – 1802,3	7,3 ± 2,8	1,7 ± 0,2 (1,60)	20,6 ± 5,6 (26,10)	1,9 ± 0,5
		Adapted [8]	2681	114 – 862	459,6 – 3437,5	6,6 ± 1,3	0,8 ± 0,3 (0,39)	24,4 ± 5,9 (34,03)	2,2 ± 0,5
KC/M	NPLC	Early [3]	410	114 - 173	207,5 – 714,0	1,5 ± 0,6	0	7,6 ± 2,9 (11,00)	-
		Adapted [3]	655	218 - 219	544,9 – 644,2	2,6 ± 0,2	0,9 ± 0,4 (0,62)	9,3 ± 3,6 (13,37)	-

[a] pHEP, parenchymal hepatocytes; NPLC, non-parenchymal liver cells; KC/M, Kupffer cells-macrophages. [b] LCI-monitoring periods: pHEP: early (0-24h after plating) and adapted culture (24-48h after plating); NPLC: early (0-3h after plating) and adapted culture (> 6h after plating). The number of different monitored hTCL trajectories from N=4 (pHEP) and N=2 (NPLC) independent primary cultures is given in parentheses. [1] Total number of scored video frames. Time resolution = 1 frame per minute. [2] Minimum and maximum duration of the LCI-monitoring intervals in which hTCLs were monitored. [3] Minimum and maximum cumulative distances of hTCL movement within the LCI-monitoring intervals. [4] The lowest/highest measured velocity within all LCI-experiments is given in parentheses.[5] Shown is the mean index for the highest velocity / 10.88 (i.e. the mean equivalent diameter determined for hTCL (see Table S2). Size measurements were not carried out in KC/M. Velocity values are mean ± SD.
